# Reproducible, high-yielding, biological caproate production from food waste using a single-phase anaerobic reactor system

**DOI:** 10.1186/s13068-018-1101-4

**Published:** 2018-04-11

**Authors:** Corine Orline Nzeteu, Anna Christine Trego, Florence Abram, Vincent O’Flaherty

**Affiliations:** 1 Microbial Ecology Laboratory, Microbiology, School of Natural Sciences and Ryan Institute, National University of Ireland (NUI), Galway, Ireland; 2 Microbial Communities Lab, Microbiology, School of Natural Sciences, National University of Ireland (NUI), Galway, Ireland; 3 Functional Environmental Microbiology, Microbiology, School of Natural Sciences, National University of Ireland (NUI), Galway, Ireland

**Keywords:** Food waste, Leach-bed reactor, Caproate, Hydrolysis, Fermentation, *Clostridium* sp., Electron donors

## Abstract

**Background:**

Nowadays, the vast majority of chemicals are either synthesised from fossil fuels or are extracted from agricultural commodities. However, these production approaches are not environmentally and economically sustainable, as they result in the emission of greenhouse gases and they may also compete with food production. Because of the global agreement to reduce greenhouse gas emissions, there is an urgent interest in developing alternative sustainable sources of chemicals. In recent years, organic waste streams have been investigated as attractive and sustainable feedstock alternatives. In particular, attention has recently focused on the production of caproate from mixed culture fermentation of low-grade organic residues. The current approaches for caproate synthesis from organic waste are not economically attractive, as they involve the use of two-stage anaerobic digestion systems and the supplementation of external electron donors, both of which increase its production costs. This study investigates the feasibility of producing caproate from food waste (FW) without the supplementation of external electron donors using a single-phase reactor system.

**Results:**

Replicate leach-bed reactors were operated on a semi-continuous mode at organic loading of 80 g VS FW l^−1^ and at solid retention times of 14 and 7 days. Fermentation, rather than hydrolysis, was the limiting step for caproate production. A higher caproate production yield 21.86 ± 0.57 g COD l^−1^ was achieved by diluting the inoculating leachate at the beginning of each run and by applying a leachate recirculation regime. The mixed culture batch fermentation of the FW leachate was able to generate 23 g caproate COD l^−1^ (10 g caproate l^−1^), at a maximum rate of 3 g caproate l^−1^ day^−1^ under high H_2_ pressure. Lactate served as the electron donor and carbon source for the synthesis of caproate. Microbial community analysis suggested that neither *Clostridium kluyveri* nor *Megasphaera elsdenii,* which are well-characterised caproate producers in bioreactors systems, were strongly implicated in the synthesis of caproate, but that rather *Clostridium* sp. with 99% similarity to *Ruminococcaceae bacterium CPB6* and *Clostridium* sp*. MT1* likely played key roles in the synthesis of caproate. This finding indicates that the microbial community capable of caproate synthesis could be diverse and may therefore help in maintaining a stable and robust process.

**Conclusions:**

These results indicate that future, full-scale, high-rate caproate production from carbohydrate-rich wastes, associated with biogas recovery, could be envisaged.
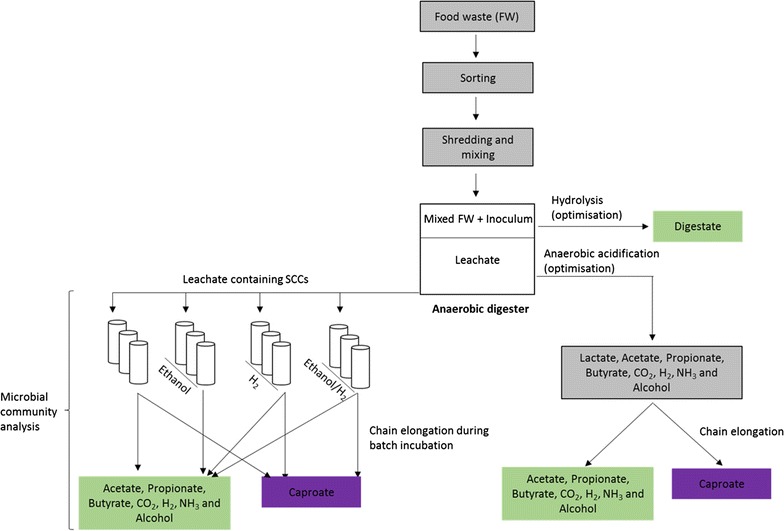

**Electronic supplementary material:**

The online version of this article (10.1186/s13068-018-1101-4) contains supplementary material, which is available to authorized users.

## Background

Anaerobic digestion (AD) of food waste (FW) is a well-established process for the sustainable production of fuels (primarily methane) [1]. Digesters processing food waste for methane production, however, suffer from instabilities especially at high organic loading rates [2]. One of the most widely reported reasons for process instability in anaerobic digesters is reactor acidification, which occurs due to the accumulation of carboxylates. These carboxylates are valuable chemicals in their own right and are more likely to be produced at high concentrations from waste with high organic content, such as FW. Recent studies have thus shifted the focus from the production of methane to the production of carboxylates, as these compounds often have a higher market value than that of methane [3]. The large-scale production of alternative valuable by-products (carboxylates) represents a new opportunity for AD technology.

Carboxylates are organic compounds containing a carboxyl group. They are classified as either short chain, containing 2–5 carbon atoms (e.g. acetate, lactate, propionate, valerate and butyrate), or medium chain, containing 6–12 carbon atoms (e.g. caproate, heptanoate and caprylate). Short chain carboxylates (SCCs) are primarily produced during the initial phase of the AD process, known as the acid-forming phase [4]. Separating them from broth has proven difficult, however, due to their high solubility in water [5, 6]. Instead, SCCs have become a platform for the synthesis of higher energy density chemicals, i.e. medium chain carboxylates (MCCs), via the process of chain elongation, which occurs via the reverse β-oxidation pathway [7–9]. This process, which has been described mainly in *Clostridium kluyveri*, is cyclic and involves the addition of acetyl-CoA molecule derived from ethanol to a carboxylate, thus elongating its carbon chain length by two carbons. Ethanol is currently the best-known electron donor for MCCs synthesis [10]. Other molecules including methanol, propanol, amino acids, pyruvate and some simple sugars have also been used as electron donors for chain elongation into MCCs [10–12]. The yields of the elongated product reported in these studies were low, however, compared to studies in which ethanol was the electron donor. Recently, Kucek et al. [13] and Zhu et al. [14] reported chain elongation into caproate using lactate, rather than ethanol, as the primary electron donor. The concentrations reported in their studies were comparable with previous studies using ethanol as an electron donor. They suggested that *n*-caproate production from lactate may be similar to the process of ethanol oxidation/reverse β-oxidation with lactate being firstly oxidised to form acetyl-CoA.

The majority of bacteria that are reported to date to be capable of chain elongation are Clostridia [10]. Of these, *C. kluyveri* has been best-studied in pure culture [15], but other bacteria, including *Eubacterium pyruvativorans* [16], *Clostridium* sp*. Bs*-*1* [17], *Ruminococcaceae bacterium CPB6* [18], and *Megasphaera elsdenii* [19, 20], have also been reported to be able to carry out chain elongation.

Fermentation of complex feedstocks by mixed microbial cultures usually generates a wide range of end products in dilute concentrations, subsequently requiring purification and concentration, resulting in costly downstream processing. Thus, to make the carboxylate platform cost-effective, the mixed culture fermentation needs to be steered towards the selective production of a single product with a maximum yield. Until now, directing mixed culture fermentation of complex feedstock towards a single and specific end product has remained challenging [21, 22].

Although a number of studies are available on carboxylate production from complex feedstock (FW and organic fraction of solid municipal waste), nearly all of them have relied on the supplementation of external electron donors [6, 23, 24]. These experimental strategies, however, increase the production cost of the MCCs, making the process less economically attractive.

The main objectives of this work were to: (1) investigate the feasibility of MCC production from FW without the addition of external electron donors; (2) evaluate the effect of external electron donors (ethanol and hydrogen) supplementation on MCC synthesis from FW fermentation liquor; and (3) characterise microbial community dynamics during MCC production.

## Methods

### Source of food waste and inoculum

Approximately, 0.5 tonnes of restaurant FW from a fresh collection was provided by Mr. Binman (Limerick, Ireland). The FW, mainly consisting of meat, tissue papers, potatoes, rice, fruit and vegetable peelings, was homogenised (by mixing) after manually removing bones, shells and other non-degradable materials. The homogenised FW was packed in bags of 5 kg and stored at − 20 °C. Prior to each experiment, a 5 kg bag of FW was defrosted slowly in the fridge before the particle size was manually reduced through shredding with scissors. Anaerobic granular sludge from a full-scale internal circulation (IC) anaerobic digester (Carbery Milk Products, Ballineen, Co Cork, Ireland) was used as the starting inoculum. The physical and chemical characteristics of the FW and starting inoculum used in this study are shown in Table 1.

**Table 1:** Characteristics of food waste and inoculum

Parameters	Food waste	Inoculum^a^
Total solid (TS)^b^ (%)	28.19 ± 2.32	9.01 ± 0.09
Volatile solid (VS)^b^ (%)	25.96 ± 2.08	7.85 ± 0.04
VS/TS (%)	92	87
tCOD (g COD g^−1^ VS)	1.45 ± 0.16	–
sCOD (g COD g^−1^ VS)	0.11 ± 0.002	–
Total hemicellulose^c^ (%)	32.58 ± 4.48	–
Total cellulose^c^ (%)	2.82 ± 0.95	–
Total protein^c^ (%)	20.69 ± 1.17	–
Total fat^c^ (%)	27.50 ± 1.45	–
pH	3.56 ± 0.3	7.46 ± 0.25

All values show standard deviation with *n* = 3

*tCOD* total chemical oxygen demand, *sCOD* soluble chemical oxygen demand

^a^ Granular sludge used only at the beginning of reactor’s run

^b^ Based on wet weight

^c^ Based on dry weight

### Reactor design

**Fig. 1:**
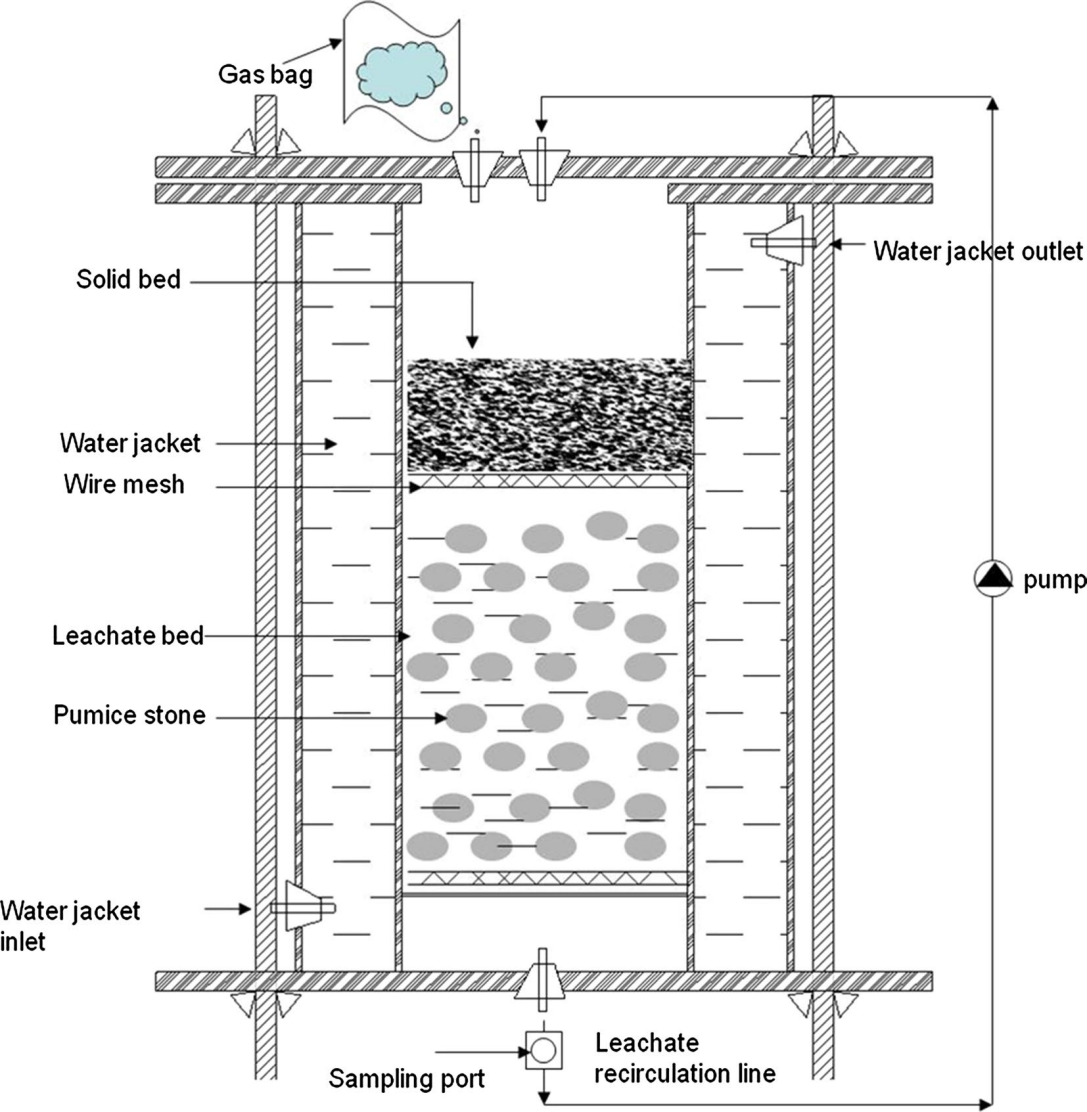
Schematic diagram of leach-bed reactor

Three leach-bed reactors (R1, R2 and R3) were operated in a semi-continuous mode with a 14-day solid retention time (SRT; period 1) and 7-day SRT (period 2). The leach-bed reactors (Fig. 1) had a total and a working volume of 6 and 3 l, respectively. Each reactor comprised two chambers (upper and lower) separated by a wire mesh. The working volume was defined as the volume of the upper chamber. In the lower chamber, a pumice stone bed containing 0.5 kg of water-washed and oven-dried pumice stone was placed above a wire mesh to facilitate the filtering of the leachate and prevent clogging of the recirculation line. All three reactors were made of acrylic column and operated at a constant temperature of 37 °C maintained by the water jacket surrounding the column. A Watson-Marlow pump (Laboratory pump with 313D rapid load flip-top pumphead) was used for leachate recirculation from the lower to the upper chamber.

### Reactor operation

The granular sludge inoculum was mixed with FW at the ratio of 0.25 (inoculum/FW) based on VS and 40 g of sodium bicarbonate (NaHCO_3_) was added. The mixture was loaded into the upper chamber above the wire mesh of each reactor. The total organic loading for each reactor was 80 g VS FW l^−1^. One litre of water was added to the lower chamber and the reactors were sealed airtight with a lid. Reactors were operated at 14-day SRT and leachate recirculation was initiated for 1 h day^−1^ at 20 ml min^−1^. The reactors were operated in a semi-continuous mode, whereby on day 14, at the end of a batch, a subsequent batch was started. Inoculation of fresh FW was carried out using digestate from the previous batch with a ratio of 0.25 (inoculum/FW). Similarly, leachate from day 14 was diluted four times and pH adjusted to 7 with NaHCO_3_, to be used as starting leachate for the subsequent batch.

### Strategies to improve caproate yield in leach-bed reactors

Different strategies were tested to improve the yield of caproate during FW degradation in leach-bed reactors (Additional file 1: Table S1). Initially, the SRT of the three leach-bed reactors previously operated at 14 days (period 1) was reduced to 7 days (period 2). Period 2 involved six consecutive phases of reactor optimisation. Initially, the operating conditions were similar to those employed during the 14-day SRT experimental phase (as described above) with the only exception that 7-day SRT was applied. After running several 7-day SRT batches (Additional file 1: Table S1), leachate recirculation was increased from once a day (phase 1) to four times a day (phase 2). A third phase consisted of reducing the concentration of volatile fatty acid (VFA) in the starting leachate from circa 15 to 6 g COD l^−1^ (by diluting 15 times the leachate from the previous batch). During phase 4, each reactor was bio-augmented with enriched cultures grown on Whatman filter paper 1 (source of cellulose), xylan (source of hemicellulose), skimmed milk (source of protein), oleate and palmitate (source of fat) as the only carbon source. Details on the development of enriched cultures and bio-augmentation are provided in the Additional file 1: Section S1–S5 and Figure S1. Phase 5 consisted of simulating the removal of VFAs in the leachate on day 2 of the retention period by replacing half of reactor leachate with water. Finally, phase 6 consisted of increasing the organic loading rate from 80 to 120 g VS l^−1^. During period 1 and 2, reactor performance was monitored by measuring the pH, VS removal and VFA production.

### Medium chain carboxylate synthesis in batch assay

To investigate the impact of electron donors (ethanol and hydrogen) on caproate synthesis from SCCs present in the leachate recovered from R2 of batch 31 (phase 6), batch fermentation assays were performed using 160 ml vials. 37 ml of leachate (pH 4.8) withdrawn from R2 on day 2 of batch 31 (phase 6) was added to each vial. The leachate pH in all the vials was adjusted to 7 by the addition of 1 N NaOH. The synthesis of MCCs was studied without (control) and with supplementation of hydrogen (H_2_), ethanol or a combination of hydrogen and ethanol (H_2_/ethanol). The initial concentrations of ethanol and acetate in all vials were 4 and 9 g l^−1^, respectively. The concentration of ethanol in ethanol and H_2_/ethanol-supplemented vials was adjusted so that ethanol was twice the concentration of acetate. A combination of H_2_/CO_2_ gas (at the ratio of 80/20) was injected at 0.5 bar of pressure for 5 s in H_2_- and H_2_/ethanol-supplemented vials.

### Chemical analysis

Soluble chemical oxygen demand (sCOD) concentrations in the leachate samples were measured according to the Standing Committee of Analysts [25]. Samples were analysed for TS and VS using standard methods [26]. Lipid, hemicellulose, cellulose and fat content of FW were determined as indicated in the Additional file 1: Section S6. Biogas composition was analysed using a gas chromatograph (GC; Varian) equipped with a glass column and a flame ionisation detector. The carrier gas was nitrogen and the flow rate was 25 ml min^−1^. Volatile fatty acid (VFA) was quantified in a Varian Saturn 2000 GC equipped with CombiPal autosampler and a flame ionisation detector. VFA separation was carried out on a BP 21, FFAP capillary column (SGE analytical science) of 30 m length, 0.25 mm internal diameter and 0.25 µm film. Helium was used as the carrier gas at a flow of 1 ml min^−1^. The GC oven temperature was programmed as follows: from 60 °C (10 s) to 110 °C (20 s) at a rate of 30 °C min^−1^; from 110 °C to 200 °C (2 min) at a rate of 10 °C min^−1^. The temperatures of the injector and detector were 250 °C and 300 °C, respectively. The volume injected was 1 µl. VFA quantification was achieved by using calibration curves of standard VFAs. Prior to GC analysis, the samples were prepared by adding orthophosphoric acid to a final concentration of 5% and centrifuging in a 2 ml safe-lock microcentrifuge tube in a fixed angle rotor at 18,000×*g* for 10 min (Eppendorf 5415 D). Lactate and ethanol assay kits (Megazyme) were used to measure the concentrations of lactate and ethanol, respectively, according to the manufacturer’s instructions.

### RNA extraction and cDNA generation

To investigate the microbial groups potentially involved in MCCs synthesis during batch experiment, 30 ml samples were withdrawn from the control, ethanol H_2_- and H_2_/ethanol- supplemented vials at day 0, 4, 6 and 11. All the samples were centrifuged at 8000×*g* for 15 min, and the pellets were used for RNA extractions using the method described by Griffiths et al. [27] with modifications (detailed in Additional file 1: Section S7). RNA purification was carried out using the TURBO DNA-free™ Kit (Ambion by Life Technology) in accordance with the manufacturer’s instructions. Complementary deoxyribonucleic acid (cDNA) was generated from DNA-free RNA samples using the superscript reverse transcriptase III (Invitrogen) following the manufacturer’s recommendations. The V4 region of bacterial and archaeal 16S rRNA was amplified from cDNA samples using the primer set 515F and 806R [28]. Each reaction consisted of a 25 µl reaction mixture containing 1 × Q5^®^ Reaction Buffer, 200 µM of each dNTPs, 0.2 µM of each primer and 2 × 10-2 U µl^−1^ Q5 High-Fidelity DNA Polymerase (New England BioLabsinc). The PCR conditions were as follows: initial denaturation at 94 °C for 30 s, 30 cycles of annealing at 50 °C for 30 s, and elongation at 72 °C for 30 s. The final extension was carried out at 72 °C for 2 min. Amplicons were subsequently purified using the NucleoSpin^®^ Gel and PCR Clean-up kit (Macherey-Nagel) in accordance with the manufacturer’s instructions. Purified amplicons from all samples were normalised to a final concentration of 20 ng µl^−1^ and sent to an external laboratory (Research and testing laboratory, Texas US) for 16S rRNA amplicon sequencing using MiSeq Illumina platform.

### Phylogenetic analysis

16S rRNA gene sequences were downloaded from the NCBI Blastn website (https://blast.ncbi.nlm.nih.gov/Blast.cgi). Phylogenetic tree was built using Mega 7 software [29] with the neighbour-joining method. The evolutionary distances were computed using the maximum composite likelihood method.

### Statistical analysis

The one-way ANOVA (analysis of variance) in Minitab 17 was used to analyse the data and test the significance of results.

### Accession number

16S rRNA sequence reads for all samples analysed within this study are available from the Sequence Read Archive database (Accession Number SRP125975).

## Results

### Hydrolysis of food waste inside leach-bed reactors

**Fig. 2:**
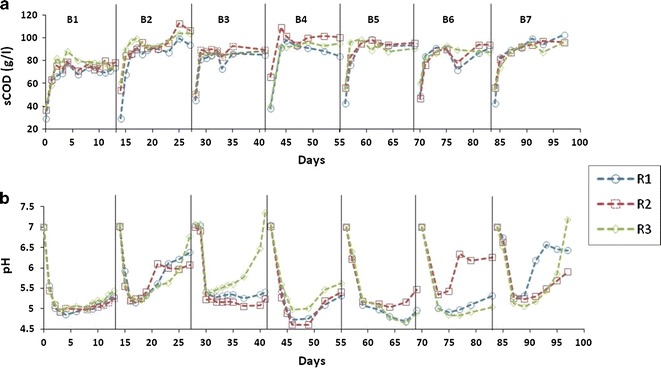
Profile of the sCOD accumulated in the leachate (**a**) and pH (**b**) during period 1. Reactors R1, R2 and R3 were operated at 14-day SRT. B1–B7: batch 1–7

**Fig. 3:**
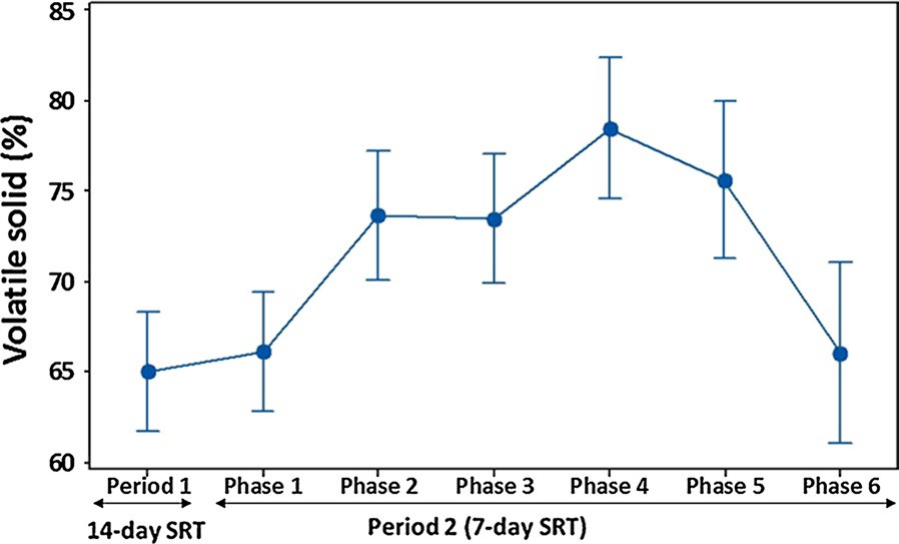
Profile of the 95% CI for the mean volatile solid (VS) reduction. VS was measured during period 1 (14-day SRT) and period 2 (7-day SRT). Number of batches for period 1 is 7 and for period 2 is 7, 6, 6, 5, 4, and 3 for phase 1–6, respectively. The bar chart for the actual VS reduction during both periods is shown on Additional file 1: Figures S5 and S6

During period 1, when the reactors were operated at a 14-day SRT, the sCOD in leachate increased rapidly during the first 2 days of the process and then remained constant until the end of the batch (Fig. 2a). The sudden initial increase of sCOD in the leachate corresponded with a sharp decrease in pH from 7 to 5 ± 0.5 (Fig. 2b). A subsequent increase in the pH of the leachate was then observed between days 7 and 13. Similar sCOD and pH profiles were reported during period 2 where 7-day SRT was applied (Additional file 1: Figure S2). The ammonia concentration in the leachate, from all reactors (period 1), increased sharply between days 1 and 3 of the batch—displaying a similar trend to the sCOD (Additional file 1: Figure S3). Analysis of the digestate at the end of batch 7 (day 14) revealed a destruction efficiency of 99, 60, 50 and 20% for hemicellulose, proteins, cellulose and fats, respectively (Additional file 1: Figure S4). This highlighted the relatively poor breakdown of fat. The VS reduction efficiency during both period 1 (Additional file 1: Figure S5) and period 2 (phase 1; Additional file 1: Figure S6) was similar, between 62 and 68%—as evidenced by the *p* value 0.71 and the overlap of the 95% confidence intervals (CI; Fig. 3). This indicated that FW degradation could be achieved within a 7-day batch process. During period 2, it was observed that increasing the leachate recirculation regime from once per day (phase 1) to four times per day (phase 2) significantly (*p* value 0.02) improved VS destruction by 10% (Fig. 3), indicating an increase in hydrolysis efficiency. The highest VS removal, between 85 and 90%, was achieved for batch 20 during phase 4, which corresponded to the period where reactors were bio-augmented (Additional file 1: Figure S6). However, the mean VS destruction achieved during phase 4 was similar (*p* value > 0.05) to that of phase 2, 3, and 5, indicating that the effect of bio-augmentation only lasted for batch 20. Increasing FW loading from 80 to 120 g VS l^−1^ during phase 6 resulted in the reduction of the VS destruction (Fig. 3).

### Production and composition of carboxylates inside leach-bed reactors

**Fig. 4:**
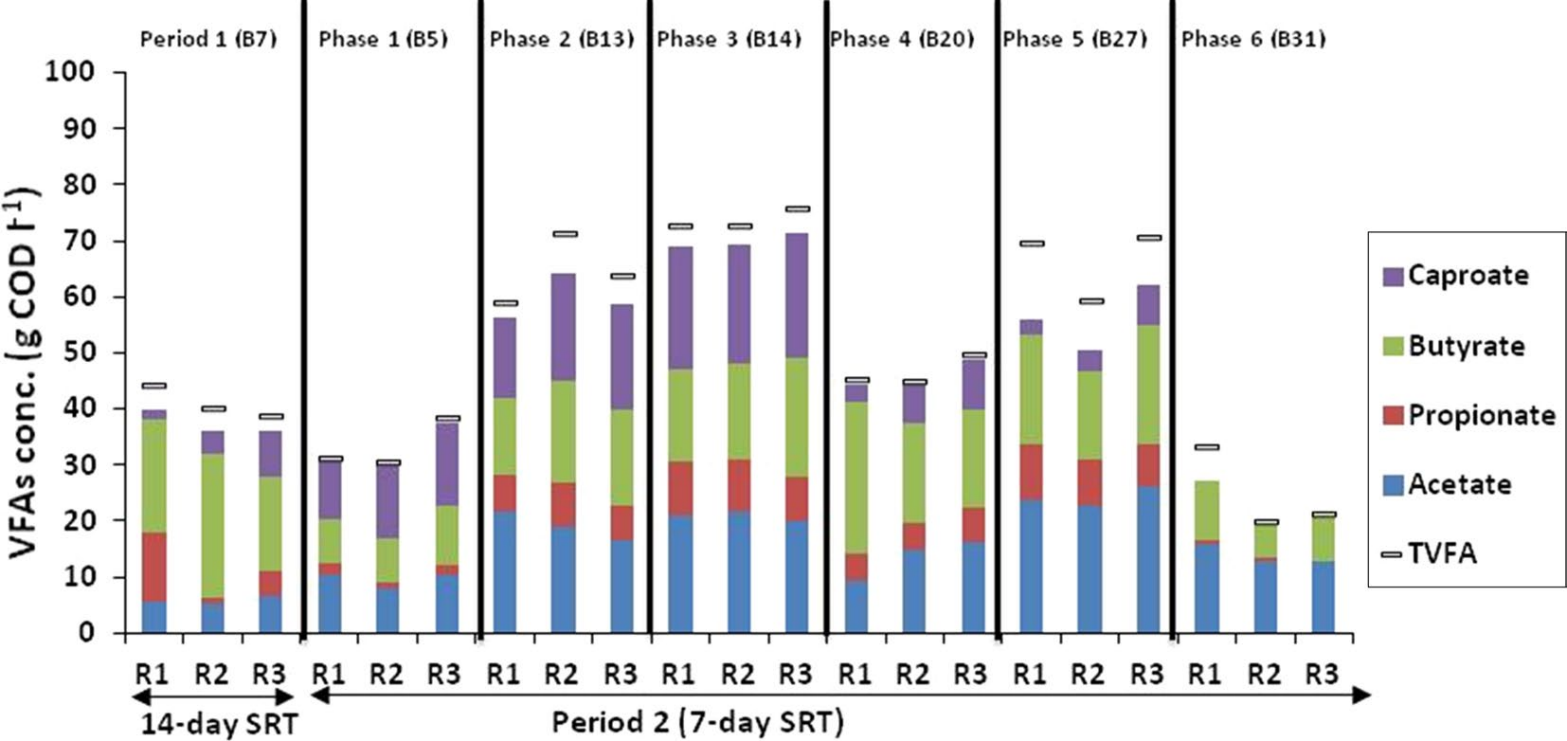
Volatile fatty acid (VFA) concentrations. VFA was measured during period 1 (14-day SRT) and period 2 (7-day SRT) of reactor (R1, R2 and R3) operation. Phase 1: leachate was recirculated on top of solid bed once a day; phase 2: leachate recirculated four times per day; phase 3: dilution of total volatile fatty acid in starting liquid; phase 4: bio-augmentation; phase 5: dilution of VFA in the leachate on day 2; phase 6: increase of loading rate. Batch (B) 7 was selected for period 1, while B5, B13, B14, B20, B27 and B31 were selected for phase 1–6, respectively

The total VFA yield produced during period 1 was 40.94 ± 2.77 g COD l^−1^ and during phase 1–6 of period 2 was 33.28 ± 4.35, 64.64 ± 6.09, 73.54 ± 1.86, 46.48 ± 2.62, 66.52 ± 6.17 and 24.70 ± 7.29 g COD l^−1^, respectively (Fig. 4). Reducing the SRT from 14 (period 1) to 7 days (phase 1 of period 2) resulted in a non-significant (*p* value 0.06) decrease of VFA yield as evidenced by the overlap of their 95% confidence intervals (Additional file 1: Figure S7). Unlike hydrolysis, which occurred within the initial 2 days of the SRT, acidification required a longer time to progress. It was observed, however, that VFA yield increased by 49% from phase 1 to phase 2 associated with the increase in recirculation frequency (from one to four times each day). The highest VFA concentration of 73.54 ± 1.86 g COD l^−1^ was achieved in phase 3, during which a combination of frequent leachate recirculation and dilution of VFA in the starting liquid (inoculating leachate) were applied. However, the one-way ANOVA revealed that the 95% confidence interval of the mean VFA concentrations during phase 2, 3 and 5 overlapped to a greater extent (Additional file 1: Figure S7) and were not significantly different (*p* value 0.16). During the period of high VFA and caproate production (phase 2 and 3), maximum acetate production was achieved within the initial 3 days of the 7-day SRT (Additional file 1: Figure S8A). On the other hand, propionate, butyrate and caproate production appeared to be mainly produced from day 3 to day 6 (Additional file 1: Figure S8B–D).

The lowest VFA yield of 24.70 ± 7.29 g COD l^−1^ was observed during phase 6, when the loading concentration was increased from 80 to 120 g VS l^−1^ (Fig. 4). The main VFA identified in reactor leachates during periods 1 and 2 included acetate, propionate, isobutyrate, butyrate, isovalerate, valerate and caproate, with acetate, butyrate and caproate representing around 80% of the accumulated VFAs. Caproate (an MCC) concentrations were among the highest during phases 1–3. The mean caproate, butyrate and acetate concentrations were similar as indicated by the *p* value 0.16. The order of VFA yield during phase 1–3 was as follows: caproate ≥ acetate ≥ butyrate > propionate. Although the highest concentration of caproate (21.86 ± 0.58 g COD l^−1^) was obtained during phase 3, the fraction of caproate as a % of total VFA (38.25 ± 4.30%) was highest during phase 1 (Table 2). Phases 4–6 yielded butyrate and acetate as the most predominant VFA. Caproate was not detected in reactor leachates during phase 6.

**Table 2:** Percentage of dominant VFAs produced in leach-bed reactors during period 1 and 2

VFA (%)	Period 1	Period 2					
		Phase 1	Phase 2	Phase 3	Phase 4	Phase 5	Phase 6
Acetate	14.2 ± 2.7	29.0 ± 4.0	29.7 ± 6.0	28.4 ± 1.8	28.8 ± 6.7	36.5 ± 2.0	57.5 ± 8.5
Propionate	14.1 ± 13.3	4.4 ± 1.2	10.8 ± 0.7	12.1 ± 1.7	11.3 ± 1.2	13.1 ± 1.9	2.0 ± 1.3
Butyrate	51.0 ± 11.2	26.5 ± 1.1	25.0 ± 1.8	24.8 ± 3.0	45.0 ± 13.6	28.1 ± 2.0	32.8 ± 3.3
Caproate	11.7 ± 8.6	38.3 ± 4.3	27.2 ± 2.6	29.7 ± 0.4	13.4 ± 5.8	6.7 ± 3.1	NA

*NA* not available because value was below detection limit

All values show standard deviation with *n* = 3

During phases 2 and 3 which corresponded to high caproate production, lactate concentration increased substantially within the initial 2 days of the 7-day SRT and subsequently decreased until the end of the retention period (Additional file 1: Figure S9). A similar trend was also observed during period 1 corresponding to 14-day SRT (Additional file 1: Figure S9).

During phases 1–4, the pH of the three reactors dropped within 3 days of incubation from an average of 7.5 to 5.2 and subsequently increased to within a range of 5.8–6.8 towards the end of the incubation period (Additional file 1: Figure S2B). The initial drop in pH was quite pronounced for phases 5 and 6, with values below five recorded within day 1 of incubation. The temporal variation in VFA production during phases 2 and 3 revealed that caproate was mainly produced towards the end of the incubation period, when pH values were above 5.5 (Additional file 1: Figure S2B). It was observed that caproate production completely ceased during phase 6, likely due to the low pH (< 5).

The fraction of methane in the biogas generated during all the phases was below 6% (Additional file 1: Table S2), indicating that methanogenesis was likely inhibited.

### Effect of external hydrogen and ethanol on MCC production

**Fig. 5:**
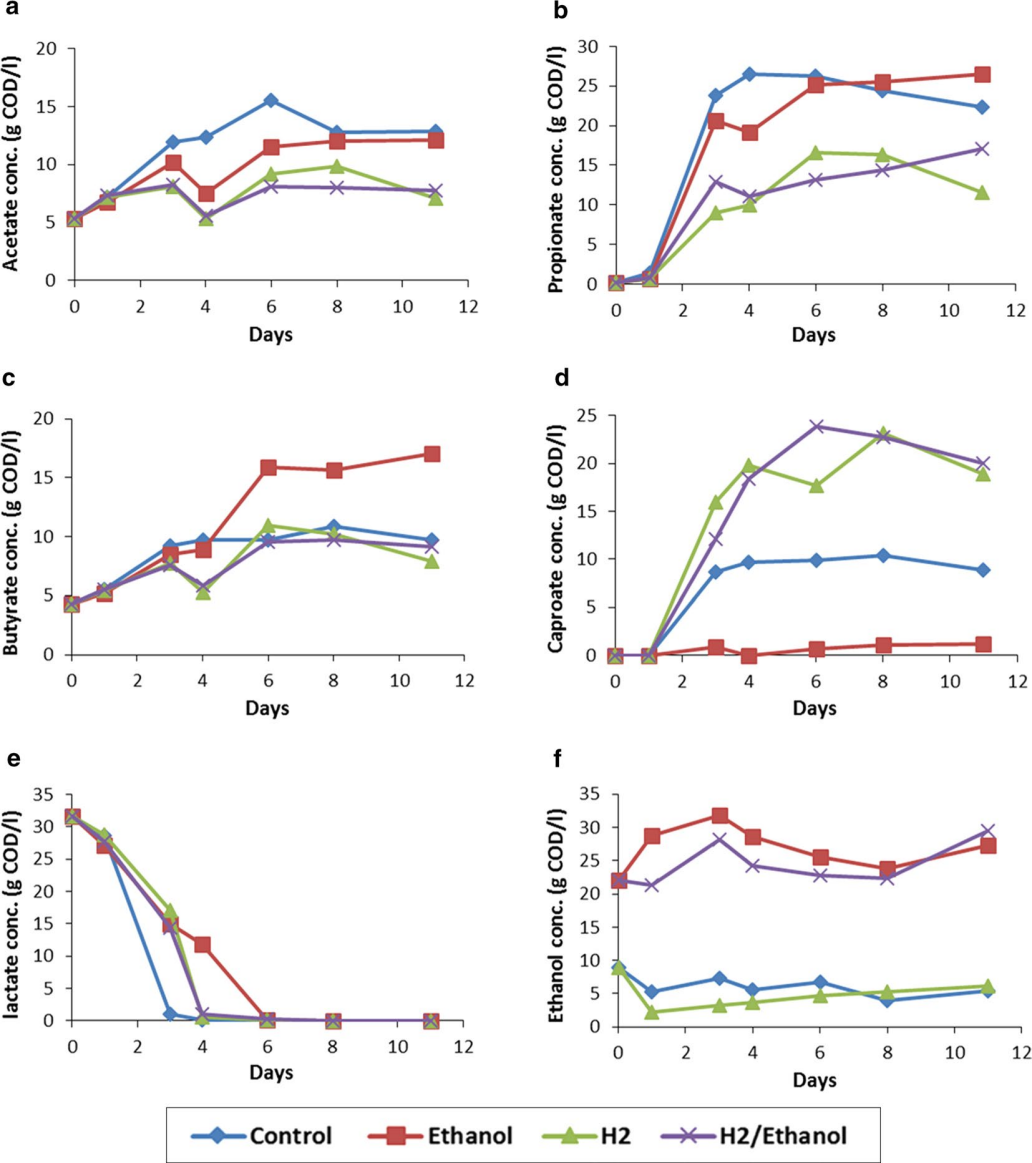
Carboxylate production from food waste fermentation liquid during batch experiments. Acetate (**a**), propionate (**b**), butyrate (**c**), caproate (**d**), lactate (**e**) and ethanol (**f**) concentrations in control, ethanol, hydrogen (H_2_) and H_2_/ethanol-supplemented vials

Batch experiments using leachate from R2 on day 2 (phase 6, batch 31) were performed to assess the impact of hydrogen and ethanol supplementation on MCCs synthesis. The concentration of lactate, acetate, ethanol and butyrate which are potential building blocks for caproate synthesis were 29.40, 5.28, 4.28 and 8.98 g COD l^−1^, respectively, in R2 on day 2. Caproate was produced in the control, H_2_- and H_2_/ethanol-supplemented vials, but was not produced in vials supplemented only with ethanol (Fig. 5d). In the H_2_- and H_2_/ethanol-supplemented vials, caproate yields were the highest, reaching 23.16 and 22.75 g COD l^−1^ after 8 days of incubation, respectively (Fig. 5d and Additional file 1: Figure S10). In the control vials, caproate production was, however, much lower (10 g COD l^−1^) and propionate was the carboxylate produced at highest concentrations (Fig. 5b). In ethanol-supplemented vials, propionate and butyrate were the main carboxylates produced (Fig. 5b and c). Lactate was completely depleted by day 3 of the incubation in the control vials, whereas, in H_2_- and H_2_/ethanol-supplemented vials complete lactate depletion had only occurred on day 4 (Fig. 5e). The lowest lactate removal rate was observed in ethanol-supplemented vials (which required 6 days). Ethanol concentrations, however, had slightly increased in all the vials by the end of the incubation period (Fig. 5f). The sCOD in H_2_-supplemented vials (where the highest caproate synthesis occurred) was relatively constant (84.41 ± 0.77 g l^−1^) throughout the incubation period, indicating that carbon mass was not lost to methane production. Methane gas could not be detected in the headspace of all the vials throughout the incubation period. The carboxylate and ethanol/sCOD ratio in vials supplemented with H_2_ was 60.13 and 76.65%, on days 0 and 8, respectively. This indicated the presence of other soluble, but unfermented molecules in the liquor in higher concentrations on day 0 than day 8.

### Microbial community composition

**Fig. 6:**
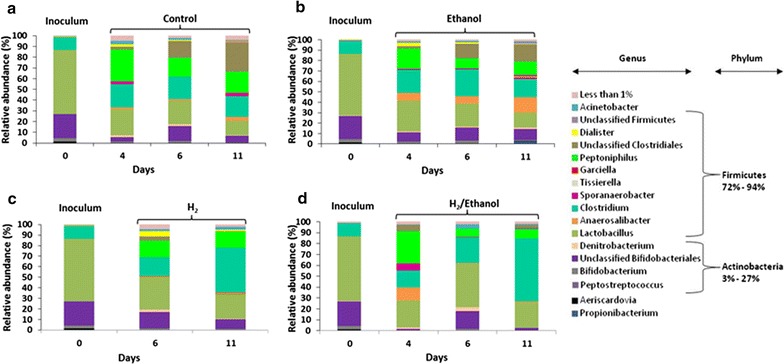
Taxonomic classification of the 16S rRNA sequences showing microbial groups potentially involved in caproate production. Sequences were retrieved from Illumina MiSeq sequencing of cDNA samples generated from: **a** control, **b** ethanol, **c** hydrogen (H_2_) and **d** hydrogen and ethanol (H_2_/ethanol)-supplemented vials during batch experiments using leach-bed reactor leachate

The microbial community dynamics in the control, ethanol, H_2_- and H_2_/ethanol-supplemented vials were investigated by means of 16S rRNA profiling from cDNA samples. In all vials, Firmicutes and Actinobacteria were the two dominant phyla based on the sequence data, representing 72–94, and 3–27% of the entire community, respectively (Fig. 6). *Clostridium* constituted 12% of microbial groups present in the inoculum; however, their relative abundance significantly increased in H_2_- and H_2_/ethanol-supplemented vials (where high caproate yield was achieved) to between 42 and 58% of sequences, within 11 days of incubation, respectively. *Clostridium* was the dominant group in H_2_-and H_2_/ethanol-supplemented vials on day 11, while in ethanol-supplemented vials, its relative abundance was comparable to the starting inoculum. Microbial community members affiliated with *Clostridium* in all vials were identified as *Clostridium cochlearium* and *Clostridium* sp. (Additional file 1: Figure S11). In H_2_-and H_2_/ethanol-supplemented vials (high caproate yield), *Clostridium* sp. represented 66 and 88% of the entire *Clostridium* group, while *C. cochlearium* only made up 34 and 12% of the genus on day 11 (Additional file 1: Figure S11). In high yield, caproate-producing vials, several OTU were identified as member of *Clostridium* sp. Among these OTU, the highest observed relative abundance was 73 and 64% for OTU 456 (H_2_-supplemented vials) and OTU 419 (H_2_/ethanol-supplemented vials), respectively. Based on 16S rRNA gene sequence analysis, OTU 419 and OTU 456 cluster with *Clostridium* sp. MT1 and *Ruminococcaceae* bacterium CPB (Additional file 1: Figure S12).

The relative abundance of *C. cochlearium* in all the vials was comparable throughout the incubation period. The change in the relative abundance of the other major microbial groups present in the inoculum (*Lactobacillus*, *Bifidobateriales* and *Peptoniphilus*) was similar in all vials including the ethanol-supplemented vials, where caproate synthesis was not detected. *Clostridiales* relative abundance mainly increased in the control and ethanol-supplemented vials, while *Anaerosolibacter* were almost specific to the ethanol-supplemented vials. Methanogenic archaea and syntrophic bacteria, which are the potential competitors to caproate-producing bacteria, especially at pH 7, were not detected in any vials, indicating that they were likely not present in the inoculum (sourced from leach-bed reactors).

## Discussion

### Bio-augmentation and high recirculation rate enhanced food waste hydrolysis

In many bioprocesses, including MCC production, the hydrolysis of complex substrates, such as those found in FW, is often the rate limiting step. Increased rates of hydrolysis are a prerequisite for achieving high yields of a desired end product from fermentation [30]. The present study measured simultaneous increases in sCOD concentration and reductions of VS to assess the rate and extent of hydrolysis. The time to achieve maximum sCOD yield (2 days) reported in this study is low compared to the 3–5 days commonly reported in studies where similar feedstock, inoculum/FW ratio and temperature were used [31–34]. The leach-bed reactor configuration adopted in this study was clearly beneficial in providing a suitable environment for hydrolytic bacteria. In the leach-bed reactor configuration, hydrolysis was accelerated by removing the soluble monomers formed in the solid bed through leachate recirculation, which creates a mixing effect likely distributing bacteria and enzymes throughout the biomass [35, 36]. By contrast with other studies, in which the recirculated liquid was either water or leachate from methanogenic reactors [35, 37–39], the present study recirculated leachate from the solid bed (within the same reactor) to prevent loss of enzymes and microbes and thus to promote rapid hydrolysis. Moreover, a higher leachate recirculation frequency (from once per day to four times per day) also contributed to enhanced FW hydrolysis. Similar findings were reported by Yesil et al. [36].

Hydrolysis of the FW was also enhanced using a bio-augmentation strategy in which hemicellulose-, cellulose-, protein- and fat-degrading mixed cultures were added to each reactor. Bio-augmentation with hydrolytic bacteria has been reported to be beneficial in several previous studies. Indeed, Cirne et al. [40] reported on the increased extent of lipid hydrolysis using bio-augmentation with an anaerobic lipolytic bacterium, isolated from bovine rumen, during the AD of lipid-rich restaurant waste. Degradation of manure fibres was also improved by using a hemicellulose degrading bacterium as a supplement [41]. Furthermore, Nielsen et al. [42] demonstrated that reactor bio-augmentation with cellulose- and hemicellulose-degrading bacteria improved the biodegradation of cattle manure. Although bio-augmentation strategies appear to be an inexpensive and simple strategy for improving hydrolysis, the ability of the enriched microorganisms to adapt to a new environment and establish within reactor systems is still perceived as poor. In the present study, the drop in VS destruction efficiency following bio-augmentation can likely be attributed to the inability of the enriched microbes to survive in the reactor environment.

The high VS removal achieved for batch 20 (phase 4, Additional file 1: Figure S6) did not directly translate into a high VFA yield. Indeed, the VFA concentrations obtained in phase 4 were 1.6 times lower than those obtained in phase 3. Yet, the opposite was observed for VS destruction, which was higher for phase 4 than phase 3. This suggests that high hydrolysis activity due to the bio-augmentation might have negatively impacted acidification. The VFA yields and VS destruction achieved during phase 6 (high loading) were the lowest on average at 24.70 g COD l^−1^ and 66%, respectively. This was likely due to an initial rapid release of soluble particulates, which contributed to the decrease in pH to values below 5—inhibiting hydrolysis and acidification. In the study of Doğan and Demirer [33], on the acidification of the organic fraction of municipal solid waste, low solubilisation efficiency was observed due to the effect of low pH on hydrolysis. The high VFA yields and VS destruction obtained in phase 5, despite the low pH (≤ 5), were attributed to VFA removal from the leachate (by dilution with distilled water). This might have contributed to acid removal and the increase in the buffering capacity—evidenced by the pH increase after leachate dilution.

### Carboxylate production is influenced by recirculation rate and dilution of the inoculating leachate

The 55% increase in VFA yield achieved during phase 3 indicated that acidification could be optimised through a combination of high leachate recirculation (4 times 1 h day^−1^ at 20 ml min^−1^) and low initial VFA concentrations (6 g COD l^−1^) in the inoculating leachate. Similar findings were reported in studies by Yesil et al. [36] and Cavdar et al. [38] on the anaerobic acidification of municipal solid waste. The operating conditions applied in phase 3 appeared optimal for the production of VFA with caproate as one of the predominant VFAs. This was an unexpected finding, as caproate has never previously been reported to be among the highest VFA constituent from direct (without addition of external electron donor) fermentation of FW. This study is the first to report high concentrations of caproate, 21.86 ± 0.57 g COD l^−1^ (10 ± 0.26 g l^−1^), from single-phase anaerobic processing of solid organic waste, without the supplementation of external electron donors under a 7-day SRT regime. Grootscholten et al. [24] reported a slightly higher caproate yield of 12.6 g l^−1^ using a two-phase anaerobic system processing municipal solid waste, but required additional ethanol supplementation. In most studies on single-phase anaerobic acidification of solid organic waste, the top three VFAs reported are acetate, propionate and butyrate [4, 32, 36, 38].

Previous studies have suggested that the optimal range pH for increased hydrolytic–acidogenic activity is between 5.5 and 6.42 [43]. Here, we observed that at a pH below 5.5, the rate of caproate formation was reduced, and its production completely inhibited below pH 5. VFA removal from the leachate (by dilution) led to increased short chain VFA production. In phase 5, when half of the leachate was replaced with water, acetate was the predominant VFA produced, followed by butyrate and propionate. Caproate concentrations remained low during phase 5, despite an increase in pH, suggesting that the removal of VFA from the leachate might have reduced the concentrations of the precursor molecules for the synthesis of caproate. Given the fact that lactate was mainly produced in the leach-bed reactors during the initial 2 days of the retention time, it was believed that the removal of half of the leachate on day 2 might have reduced the concentration of lactate which was likely the precursor for caproate synthesis. The use of lactate in the synthesis of caproate was evidenced by the fact that the period of lactate depletion correlated with the period of high caproate production.

### Successful production of caproate as the highest food waste fermentation product

This study demonstrated the feasibility of producing caproate as the main MCC constituent from anaerobic processing of FW. The concentration and production rate of caproate, notably 10 g l^−1^ and 3 g l^−1^ day^−1^, obtained from the primary fermentation broth using H_2_ supplementation was higher than previously reported. In the study of Steinbusch et al. [6], mixed culture fermentation of acetate with ethanol, hydrogen, or a combination of both was used to promote the accumulation of caproate. They reported concentrations of 8.17 g caproate l^−1^ and a maximum production rate of 0.50 g l^−1^ day^−1^ caproate per day. Similarly, a work by Ding et al. [44] reported caproate concentrations of 11–23 mM (1.3–2.7 g l^−1^) produced by mixed culture fermentation of glucose. In the research of Agler et al. [8], inline extraction was used to enhance the production of caproate from chain elongation of ethanol. The authors reported a caproate production rate of 108.3 mM C per day (2.1 g l^−1^ day^−1^). Unlike the aforementioned studies, caproate was not synthesised in the ethanol-supplemented vials in our study, indicating that ethanol was not the preferred electron donor for the synthesis of caproate. Ethanol oxidation, coupled with chain elongation, is the most commonly reported pathway for caproate synthesis. This process has mainly been investigated in *C. kluyveri*. However, in the present study, *C. kluyveri* 16S rRNA transcripts were not detected, suggesting that their growth was limited or they were likely not present in the inoculum. Furthermore, the lack of caproate synthesis in ethanol-supplemented vials indicated that higher ethanol concentration was likely inhibitory to bacteria responsible for chain elongation processes in the present study. In the control vials, after 3 days of incubation, lactate could no longer be detected, while caproate production reached its maximum. This observation implies that lactate was a critical electron donor for the formation of caproate. Similarly, the depletion of lactate appeared to correlate with the production of caproate in H_2_- and H_2_/ethanol-supplemented vials. Capraote formation from lactate was observed in the study by Zhu et al. [14]. The authors suggested that n-caproate production from lactate may share similarities with caproate formation from ethanol (oxidation/reverse β-oxidation). Lactate, rather than ethanol, is oxidised to acetate, which elongates with a second lactate molecule to form butyrate. Butyrate then elongates with a third lactate molecule forming caproate. This process consumes 3 mol of lactate to form 1 mol of caproate. Energy, acetyl-CoA and reducing equivalent are all provided during the oxidation of lactate. The overall reaction describing caproate synthesis from lactate is illustrated through the following equation:

$$ 3 {\text{CH}}_{ 3} {\text{CHOHCOO}}^{ - } \, + \, 2 {\text{H}}^{ + } \, \to \,{\text{CH}}_{ 3} \left( {{\text{CH}}_{ 2} } \right)_{ 4}  {\text{COO}}^{ - } \, + \, 2 {\text{H}}_{ 2} \, + \,{\text{H}}_{ 2} {\text{O}}\, + \,{\text{CO}}_{ 2} ; \, \Delta {\text{G}}0^{\prime} \, = \, - \, 1 2 3. 1 {\text{kJ}} {\text{mol}}^{ - 1} . $$

In the H_2_-supplemented vials, 23 g COD l^−1^ (10 g l^−1^) caproate was produced from 32 g COD l^−1^ (30 g l^−1^) lactate, meaning that 3.3 × 10^−3^ mol of caproate was synthesised from 10^−2^ mol of lactate. This agrees with the stoichiometry of the proposed chemical equation for caproate synthesis. Therefore, it can be deduced that maximum caproate selectivity, based on the consumed mole of lactate and produced mole of caproate, was 100% in the H_2_-supplemented vials. However, this selectivity was lower in the control vials (45%) for which propionate, acetate and butyrate were the main carboxylates produced. Since the only difference between control vials and H_2_-supplemented vials (from which a high caproate yield was achieved) was the supplementation of H_2_, it was deduced that high hydrogen partial pressure is necessary to achieve high caproate selectivity. The metabolic switch from caproate synthesis to other carboxylates might be possibly explained by the decline of the pre-existing H_2_ partial pressure in the control vials. This may have caused a decrease in the total energy available for the chain elongation pathway and led to the activation of more energetically favourable pathways [45]. In the control vials, some lactate was likely reduced to propionate using the acrylate pathway; this process is coupled to the production of acetate [7]. Therefore, it appears that during the chain elongation process of lactate to caproate, supplementing H_2_ could contribute to the provision of an unlimited source of reducing power [46], thereby facilitating the complete oxidation of lactate to caproate. The caproate yields of 23.41 g l^−1^ reported by Zhu et al. [14] were more than double the yields reported in the present study; however, their maximum production rate of 2.97 g l^−1^ day^−1^ was comparable to ours (3 g l^−1^). Moreover, it is worth noting that the concentration of caproate precursor, i.e. lactate, provided in the study of Zhu et al. [14] was also double that of the present study (65 g l^−1^ vs 32 g l^−1^).

### Microbial community in MCC producing vials dominated by *Clostridium* sp.

Members of the Firmicutes were likely responsible for the synthesis of caproate from lactate as they represented 72–94% of the entire community based on 16S rRNA sequencing. Moreover, the vast majority of bacteria that have been so far identified as MCC producers were found in the phylum Firmicutes [10]. The apparent increase in the relative abundance of *Peptoniphilus* in all the vials, including the ethanol-supplemented vials (where caproate was not produced), indicated that they were likely not involved in the production of caproate. *Peptoniphilus* is known to convert peptone to butyrate [47, 48]. Thus, *Peptoniphilus* were likely responsible for the metabolism of peptone (resulting from the protein breakdown during digestion) present in the vials. Similarly, the relative abundance of *Lactobacillus* decreased considerably in all the vials during the incubation period. The best-known function of *Lactobacillus* is to ferment simple sugars (e.g. glucose) to lactate. In this study, lactate was reported as the main precursor for caproate synthesis. Therefore, it could be concluded that *Lactobacillus* contributed indirectly to caproate synthesis. The percentage increase in the relative abundance of *C. cochlearium* was comparable across caproate-producing vials and non-caproate-producing vials, suggesting that they did not play an important role in the synthesis of caproate. *C. cochlearium* have been reported to be likely involved in the conversion of amino acids into butyrate [49]. *Clostridium* sp. was the only group whose relative abundance increased considerably in caproate-producing vials. Furthermore, they were the most abundant microbial group in caproate-producing vials at the end of the incubation period (day 11). Therefore, *Clostridium* sp. may play an important role in the use of lactate and in the production of caproate. The corresponding OTUs, OTU 419 and OTU 456, were 99% similar to *Clostridium* sp. MT1 and *Ruminococcaceae bacterium CPB6*, respectively. In a recent study, caproate production from lactate was demonstrated using a pure culture of *Ruminococcaceae bacterium CPB6* [18]. However, further investigation to characterise the *Clostridium* sp. cluster and elucidate their biochemistry would be required in future work. In several previous studies, *Clostridium* species have been found to be responsible for caproate production [14, 44]. *C. kluyveri* have been reported as the best-known species involved in the production of caproate from ethanol and acetate [6, 45]. However in the present study, *C. kluyveri* were not identified despite the availability of ethanol and acetate. This might suggest that using ethanol as the electron donor was perhaps not the preferred route in the presence of lactate. Surprisingly, *M. elsdenii*, previously known to be able to convert lactate to caproate, was not detected within the analysed microbiome.

In this study, the long exposure to high carboxylate concentrations during acidification in the leach-bed reactors completely inhibited the methanogenic community. Methanogens were not detected during batch experiments carried out at pH 7. In previous studies, methanogens were inhibited either by applying periodic heat shock of the inoculum [44] or by adding bromoethane sulphonic acid [6]. The former is energy demanding and additionally may also eliminate some important microbial groups that are not spore-forming bacteria. The latter is generally expensive and might negatively impact operational costs [7]. Therefore, any inhibitory effects on methanogen exposure to high concentrations of VFAs needs to be further investigated.

## Conclusions

In this study, for the first time, we demonstrated the feasibility of stabilising FW, while at the same time producing caproate as one of the highest fermentation products in a one-stage reactor system without the supplementation of external electron donors. Parameters including leach-bed reactor configuration (in which the solid bed is separated from the liquid bed) and frequent leachate recirculation contributed to creating favourable conditions for the hydrolysis of FW which occurred within 2 days. The optimal conditions to achieve high caproate yields (21.86 ± 0.58 g COD l^−1^) in leach-bed reactor were as follows: organic loading = 80 g VS FW l^−1^; T = 37 °C; SRT = 7 days; leachate recirculation regime = 4 times 1 h day^−1^ at 20 ml min^−1^ and VFA yield in inoculating leachate = 6 g COD l^−1^. We demonstrated that caproate production was not directly impacted by the availability of VFAs (acetate, propionate and butyrate). Instead, lactate and hydrogen appear to be the precursors for the synthesis of caproate. Therefore, the mixed culture fermentation of food waste for MCC production using leach-bed reactor configuration is well compatible with a second phase biogas recovery. *Clostridium* sp. were likely involved in the formation of caproate. The findings of this study have strong application potential, specifically in the design of a process that will allow for the continuous and sustainable industrial production of caproate, not only from food waste but also from other low-cost wastes with high carbohydrate content.

## Additional file

**Additional file 1.** **Table S1.** Strategies applied to improve caproate production from food waste. **Section S1.** Enrichment culture assays. **Section S2.** Substrate utilisation assays using enriched cultures. **Section S3.** Enrichment culture processes successfully selected for cellulose and hemicellulose degraders. **Section S4.** *Bacteroides graminisolvens* and *Porphyromonodaceas* are implicated in cellulose and hemicellulose hydrolysis, respectively. **Section S5.** Bio-augmentation Assay. **Section S6.** Characterisation of the food waste and digestate. **Figure S1.** Microbial communities profiling from the granular seed sludge, whatman filter paper 1 (WP) and xylan enrichment cultures (18th generation) assigned from the 16S rRNA gene sequencing from DNA samples. **Section S7.** Ribonucleic acid extraction. **Figure S2.** Profile of soluble chemical oxygen demand (sCOD) (A) and pH (B) in the triplicate reactors R1, R2 and R3 operated at 7-day SRT for each batch (period 2). Phase 1: leachate was recirculated on top of solid bed once per day; Phase 2: leachate recirculated four times per day; Phase 3: dilution of VFAs in starting liquid; Phase 4: bio-augmentation; Phase 5: dilution of VFAs in the leachate on day 2; Phase 6: increase of loading rate. Batch (B) 5, 13, 14, 20, 27 and 31 were selected to represent phase 2, 3, 4, 5 and 6. **Figure S3.** Profile of ammonia concentration in leachate samples during 14-day SRT. **Figure S4.** Degradation efficiency of the major components of restaurant food waste in R1, R2 and R3 during batch 7 (14-day SRT). **Figure S5.** Volatile solid (VS) reductions from the restaurant food waste in R1, R2 and R3 operated at SRT of 14 days for each batch. **Figure S6.** Volatile solid (VS) reductions from the restaurant food waste in triplicate reactors R1, R2 and R3 operated at 7-day SRT for each batch. Phase 1: leachate was recirculated on top of solid bed once a day for 45 min; Phase 2: leachate recirculated four times per day; Phase 3: dilution of VFAs in starting liquid; Phase 4: bio-augmentation; Phase 5: dilution of VFA in the leachate on day 2; Phase 6: increase of loading rate. **Figure S7.** Plot of 95% confidence interval for mean of volatile fatty acid (VFA) concentration during period 1 and period 2. **Figure S8.** Profile of individual VFA production in leach-bed reactors R1, R2 and R3 during Phase 2 and 3 (period 2). Batch (B) 13 and 14 were selected to represent phase 2 and 3 corresponding to high and low VFA concentrations in starting liquid respectively. Data at each point represent average of duplicate measurements. **Figure S9.** Profile of the lactate concentration in triplicate leach-bed rectors (R1–R3) during period 1 and 2 (phase 2 and 3). Batch (B) 7 was selected to represent period 1 corresponding to 14-day SRT. B13 and 14 were selected to represent phase 2 and 3 corresponding to high and low VFA concentrations in starting liquid respectively. Value at each point is the average of duplicate measurements. **Table S2.** Methane (CH4) fraction of biogas during period 1 (14-day SRT) and period 2 (7-day SRT) of reactor operation. Batch (B) 7 was selected to represent period 1while B5, 13, 14, 20, 27 and 31 represented period 2. **Figure S10.** Production pattern for acetate (A) propionate (B), butyrate (C), caproic (D), lactate (E) and ethanol (F) obtained in the control, ethanol, hydrogen (H2) and H2/Ethanol-supplemented vials during batch experiment using leach bed-reactor leachate. Data from the duplicate measurements are shown on the graph. **Figure S11.** Taxonomic classification at species level of the 16S rRNA sequences showing microbial groups potentially involved in caproate production. Sequences were retrieved from Illumina MiSeq sequencing of cDNA samples generated from: A) control, B) ethanol, C) hydrogen (H_2_) and D) hydrogen and ethanol (H_2_/ethanol) supplemented vials during batch experiments using leach-bed reactor leachate. **Figure S12.** Phylogenetic tree build using the neighbour-joining method. Analysis performed using 16S rRNA gene sequences. The numbers at the node represent the bootstrap values. The evolutionary distances were computed using the maximum composite likelihood method.
