# Supplementary material for: Reproducible, high-yielding, biological caproate production from food waste using a single-phase anaerobic reactor system
Source: Biotechnol Biofuels. 2018 Apr 11;11:108. doi: 10.1186/s13068-018-1101-4 (PMC5894149; doi:10.1186/s13068-018-1101-4)
Supplement: Supplementary file 1 — Additional file 1. Table S1. Strategies applied to improve caproate production from food waste. Section S1. Enrichment culture assays. Section S2. Substrate utilisation assays using enriched cultures. Section S3. Enrichment culture processes successfully selected for cellulose and hemicellulose degraders. Section S4. Bacteroides graminisolvens and Porphyromonodaceas are implicated in cellulose and hemicellulose hydrolysis, respectively. Section S5. Bio-augmentation Assay. Section S6. Characterisation of the food waste and digestate. Figure S1. Microbial communities profiling from the granular seed sludge, whatman filter paper 1 (WP) and xylan enrichment cultures (18th generation) assigned from the 16S rRNA gene sequencing from DNA samples. Section S7. Ribonucleic acid extraction. Figure S2. Profile of soluble chemical oxygen demand (sCOD) (A) and pH (B) in the triplicate reactors R1, R2 and R3 operated at 7-day SRT for each batch (period 2). Phase 1: leachate was recirculated on top of solid bed once per day; Phase 2: leachate recirculated four times per day; Phase 3: dilution of VFAs in starting liquid; Phase 4: bio-augmentation; Phase 5: dilution of VFAs in the leachate on day 2; Phase 6: increase of loading rate. Batch (B) 5, 13, 14, 20, 27 and 31 were selected to represent phase 2, 3, 4, 5 and 6. Figure S3. Profile of ammonia concentration in leachate samples during 14-day SRT. Figure S4. Degradation efficiency of the major components of restaurant food waste in R1, R2 and R3 during batch 7 (14-day SRT). Figure S5. Volatile solid (VS) reductions from the restaurant food waste in R1, R2 and R3 operated at SRT of 14 days for each batch. Figure S6. Volatile solid (VS) reductions from the restaurant food waste in triplicate reactors R1, R2 and R3 operated at 7-day SRT for each batch. Phase 1: leachate was recirculated on top of solid bed once a day for 45 min; Phase 2: leachate recirculated four times per day; Phase 3: dilution of VFAs in starting liquid; Phase 4: b [file 13068_2018_1101_MOESM1_ESM.pdf]

Table S1. Strategies applied to improve caproate production from food waste

Conditions	Period 1	Period 2					
	-	Phase 1	Phase 2	Phase 3	Phase 4	Phase 5	Phase 6
**Organic loading**	80 g VS FW l^{-1}	80 g VS FW l^{-1}	80 g VS FW l^{-1}	80 g VS FW l^{-1}	80 g VS FW l^{-1}	80 g VS FW l^{-1}	120 g VS FW l^{-1}
**SRT**	14	7	7	7	7	7	7
**Leachate recirculation regime**	1 time 1 h d^{-1} at 20 ml min^{-1}	1 times 1 h d^{-1} at 20 ml min^{-1}	4 time 1 h d^{-1} at 20 ml min^{-1}	4 time 1 h d^{-1} at 20 ml min^{-1}	4 time 1 h d^{-1} at 20 ml min^{-1}	4 time 1 h d^{-1} at 20 ml min^{-1}	4 time 1 h d^{-1} at 20 ml min^{-1}
**Reduction of carboxylate conc. in starting liquid [a]**	-	-	-	✓	✓	✓	✓
**Bio-augmentation**	-	-	-	-	✓	✓	✓
**Dilution of leachate on day 2 of SRT [b]**	-	-	-	-	-	✓	✓
**Number of batches**	7	7	6	6	5	4	3

SRT: Solid retention time; FW: food waste; VS: volatile solid; [a] leachate from previous batch was diluted 15 times and used as starting liquid; [b] half of the leachate from the liquid bed was removed and replaced with water.

Section S1. Enrichment culture assays

Enrichment cultures were developed using the following selected substrates: whatman filter paper 1 (WP), skimmed milk (SM), xylan, oleate and palmitate as cellulose, protein, hemicellulose, and fat sources respectively. Briefly, granular sludge was used to inoculate 19 ml of sterile anaerobic mineral medium [1], to which sterile stock of substrate was provided to a final concentration of 3 g l^{-1} except for oleate and palmitate provided at 1 mM. All cultures were incubated at 37°C and agitated. Substrates degradation was indirectly monitored by measuring the biogas accumulated in the headspace of the bottles. Distinct enrichment series were obtained by successive transfers of active cultures (10%) into fresh medium containing the relevant substrate.

Section S2. Substrate utilisation assays using enriched cultures

The ability of each enriched culture to metabolise the substrate more rapidly than the initial granular sludge was investigated by following the same procedure as the enrichment culture assays described above. This time, sacrificial vials were set up for the direct monitoring of substrate degradation. The protein concentration of the skimmed milk powder was determined by using the RC DC™ Protein Assay kit (BIO –RAD) which is based on the Lowry protocol.

Degradation of WP, xylan, palmitic and oleic acid were established gravimetrically through the reduction of the corresponding total solid contents.

Section S3. Enrichment culture processes successfully selected for cellulose and hemicellulose degraders

The hydrolysis constant k_h for WP (0.33 d^{-1}) and xylan (0.14 d^{-1}) were found to have improved when using enriched cultures as compared to the granular sludge for which K_h for WP and xylan were 0.13 d^{-1} and 0.07 d^{-1} respectively. The K_h for SM (0.67 d^{-1} and 0.74 d^{-1}) did not show any significant improvement likely due to the previous adaptation of the initial inoculum (granular sludge from dairy waste water facility) to similar substrate. As rates of degradation of oleate and palmitate were very slow, hydrolysis constants were found to be close to zero and were not reported.

Section S4. Bacteroides graminisolvens and Porphyromonodaceas are implicated in cellulose and hemicellulose hydrolysis, respectively

The microbial community composition from the seed sludge and the 18th generation of xylan (hemicellulose source) and WP (cellulose source) enriched cultures were established through *16S rRNA* gene sequencing on Illumina MiSeq platform. While a large number of phylotypes (114) were detected in the seed sludge only 25 and 15 phylotypes were found in the cellulose and hemicellulose enriched cultures respectively, indicating the development of specialised microbial communities (Fig S1). The dominating microorganism in the cellulose enriched culture accounting for 73.76% of the community was found to be affiliated with the genus Bacteroides. Conversely, 80% of the phylotypes in the hemicellulose enriched culture were associated to the family Porphyromonadaceae. Both Bacteroides and Porphyromonadaceae belong to the order Bacteriodales. *Clostridium* was the second largest community in both enriched cultures with relative abundance of 8.22% for cellulose and 14.51% for hemicellulose. Up to 71.24% of Bacteroides were identified as *Bacteroides graminisolvens* while none of the Porphyromonadaceae could be classified at the genus level. The relative abundance of archaea was high in the seed sludge compared to the two enriched cultures. *Methanobacterium* was the only archaea identified in the cellulose enriched cultures with a relative abundance of 7.61% while less than 1% methanogenic community was found in the hemicellulose enriched culture. The enrichment cultures were set up with the intention to enrich for hydrolysers rather that fermenters and methanogens.

Section S5. Bio-augmentation Assay

Bio-augmentation experiment was performed using the cellulose, hemicellulose, protein and fat enriched cultures. Five percent of each enriched culture obtained after several sub-culturing were mixed in 50 ml tubes and centrifuged at low speed (indicated the speed here please). The supernatants were discarded and the cells pellets were added to 500 ml bottles containing mixture of digestate (from R1, R2 and R3, day 7 of batch 18) and FW to a ratio of 0.25 (digestate/FW, on the VS basis). The operational conditions were similar to the one described for leach-bed reactors. After 7 days of incubation at 37°C, the content of each bottle was mixed with some digestate from day 7 of batch 19 and the mixture was used to inoculate reactor R1, R2 and R3 (containing fresh FW) at the ratio of 0.25 (inoculum/FW on the VS basis).

Section S6. Characterisation of the food waste and digestate

The lipid fractions of the FW and digestate were determined using the methanol-chloroform method previously described by Folch *et al.* [2]. Hemicellulose and cellulose fractions were determined using a combination of dilute and strong acid hydrolysis at high temperature. Briefly, 4 ml of 6 M sulphuric acid was added to 100 mg of dried FW or digestate. The mixture was incubated at room temperature (RT) for 30 min followed by the addition of 17.5 ml of water and autoclaving at 121°C (15 min holding). The tubes were centrifuged at 6000 × *g* for 15 min and supernatants containing hydrolysed sugar derived from hemicellulose were stored at room temperature (RT). The pellet was dried overnight at 60°C before concentrated sulphuric acid (18 M) was added. The tubes were incubated at RT for 30 min followed by addition of 18 ml water and autoclaving at 121°C (with 15 min holding). The tubes were centrifuged and the resulting supernatant containing glucose released from cellulose hydrolysis stored at RT. The resulting sugar solutions were analysed using the phenol-sulfuric method by Dubois *et al.* [3]. Dried RFW and digestate were sent to an external laboratory (Teagasc Food Research Centre, Ashtown, Dublin 15, Ireland) for protein quantification on a LECO FP328 (LECO Corp., MI, USA) Protein Analyser based on the Dumas method and according to AOAC method 992.15, 1990. On the LECO platform, total protein quantification is determined using nitrogen analysis. The sample is combusted with oxygen and the nitrogen released is then converted to protein using a conversion factor.

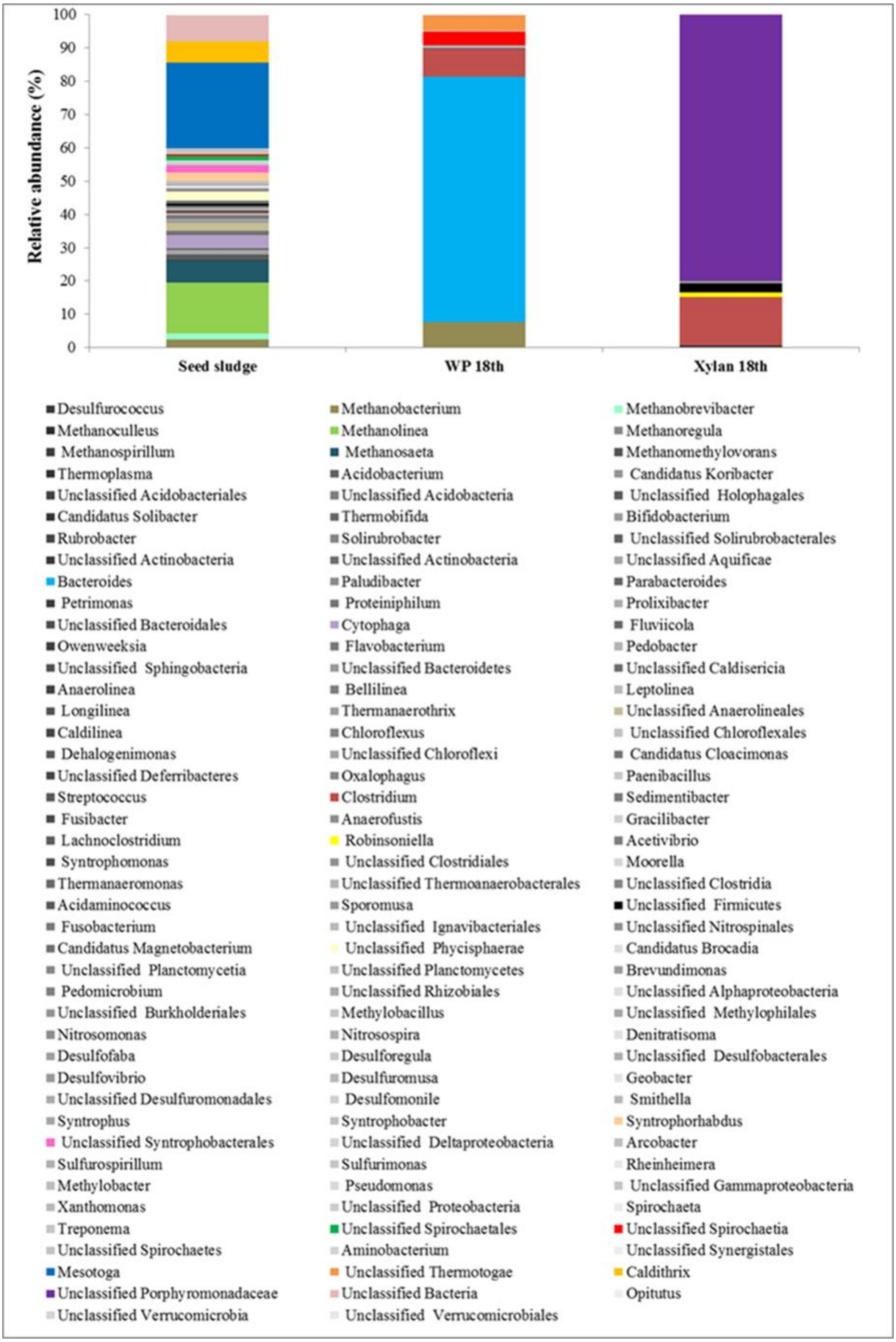

Figure S1 Microbial communities profiling from the granular seed sludge, whatman filter paper 1 (WP) and xylan enrichment cultures (18th generation) assigned from the *16S rRNA* gene sequencing from DNA samples.

Section S7. Ribonucleic acid extraction

Briefly, 0.5 ml of 10% hexadecyltrimethylammonium bromide (CTAB) extraction buffer and 0.5 ml of phenol-chloroform-isoamyl alcohol (25:24:1) were added to 2 ml Eppendorf tubes containing frozen microbial cell pellets (from -80°C). CTAB extraction buffer was prepared by mixing equal volumes of 10% (wt/vol) CTAB (Sigma, Poole, United Kingdom) in 0.7 M NaCl with 240 mM potassium phosphate buffer, pH 8.0. To each tube, 0.5 mm and 0.1 mm diameter zirconia beads (Thistle Scientific) were added and the microbial cells were lysed by bead beating for 5 min using a vortex (IKA Vortex Genius 3). The top aqueous layer were recovered after 10 min of centrifugation at 16, 000 × g and transferred to clean tubes. To remove residual phenol from previous step, 0.5 ml of chloroform-isoamyl alcohol (24:1) were added and the aqueous layer was recovered after 5 min of centrifugation at 16,000 × *g* and transferred to clean tubes. Subsequent precipitation of total nucleic acids were performed by adding two volumes of 30% (wt/vol) polyethelene glycol (PEG) 6000 (Fluka BioChemika)–1.6 M NaCl and incubated at 4°C for 2 hours followed by 20 min centrifugation at 16, 000 × g. Supernatant were carefully discarded and the pellets were washed by adding 1 ml ice cold 70% ethanol followed by 30 min centrifugation at 16,000 × g. Ethanol was completely removed and the pellets left to dry at room temperature for 2 min. Finally, the pellets containing DNA and RNA were resuspended in 50 µl RNase free water.

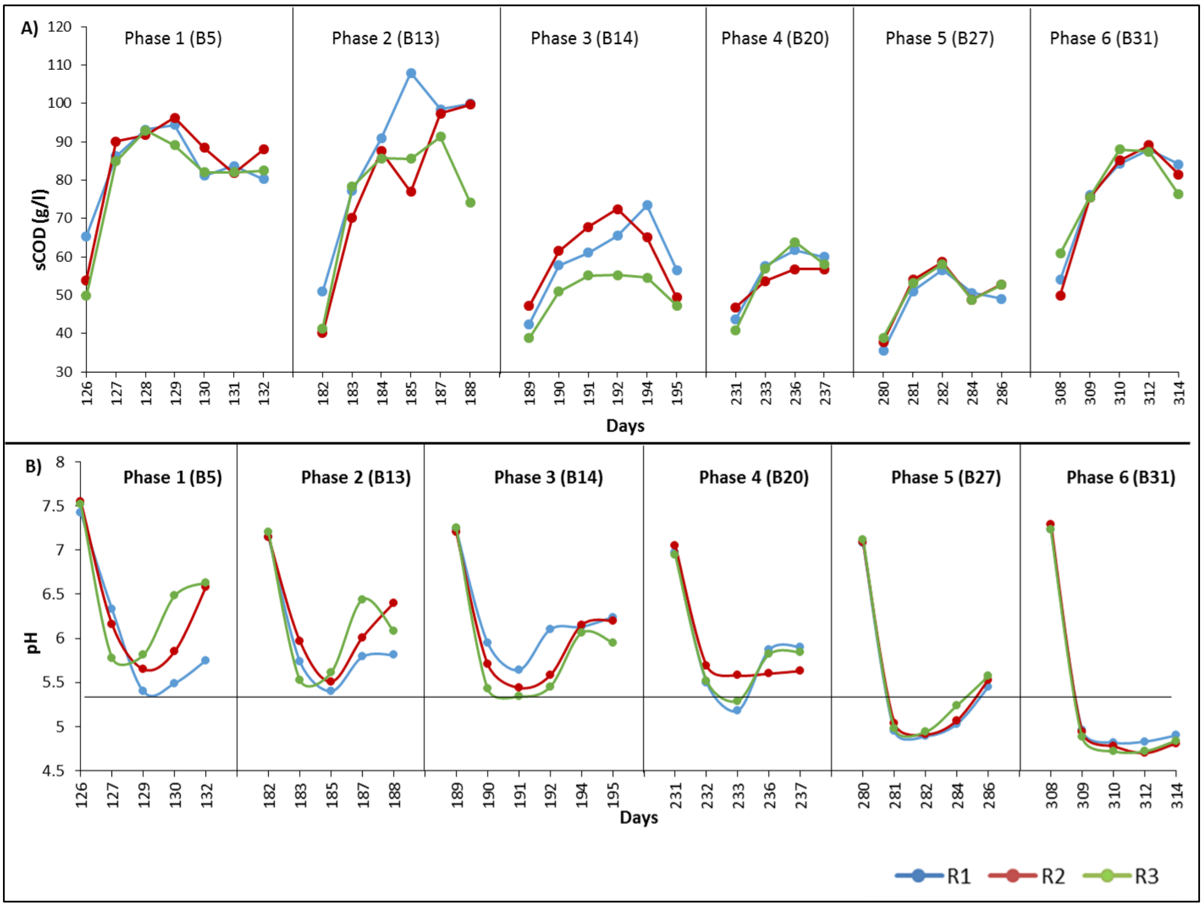

Figure S2 Profile of soluble chemical oxygen demand (sCOD) (A) and pH (B) in the triplicate reactors R1, R2 and R3 operated at 7-day SRT for each batch (period 2). Phase 1: leachate was recirculated on top of solid bed once per day; Phase 2: leachate recirculated four times per day; Phase 3: dilution of VFAs in starting liquid; Phase 4: bio-augmentation; Phase 5: dilution of VFAs in the leachate on day 2; Phase 6: increase of loading rate. Batch (B) 5, 13, 14, 20, 27 and 31 were selected to represent phase 2, 3, 4, 5 and 6.

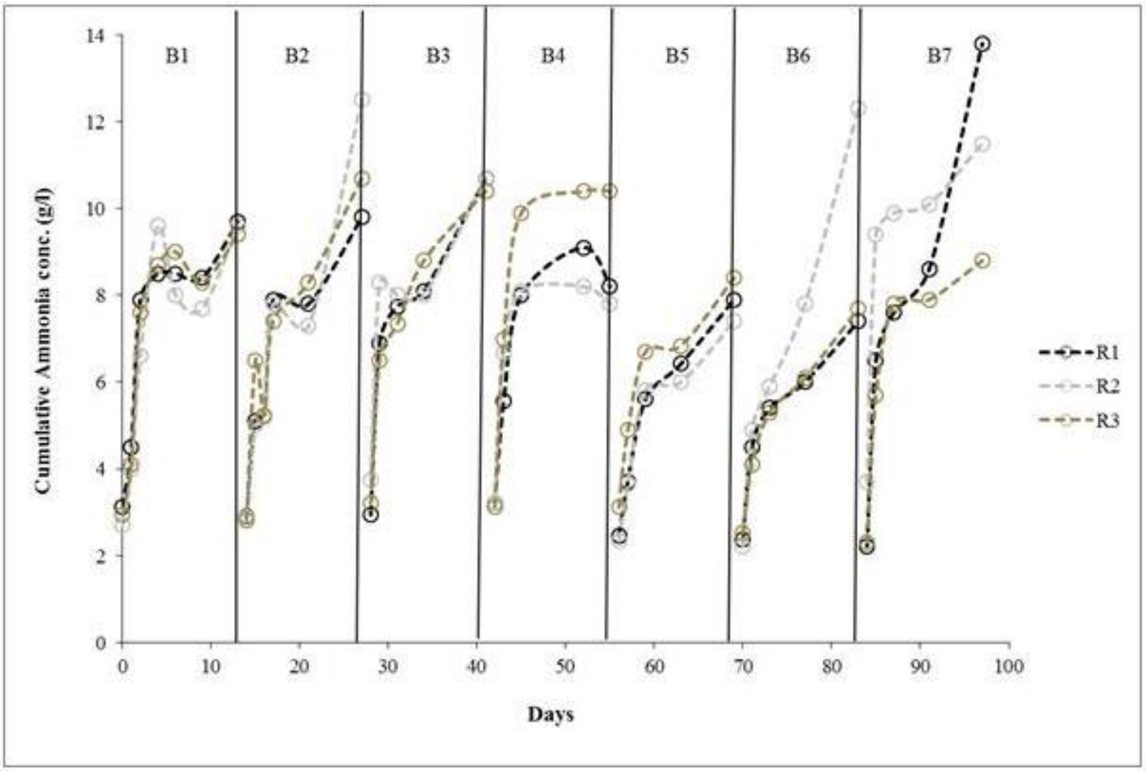

Figure S3 Profile of ammonia concentration in leachate samples during period 1 (14-day SRT). B1-B7: batch 1 to batch 7. Value at each point is the average of duplicate analysis.

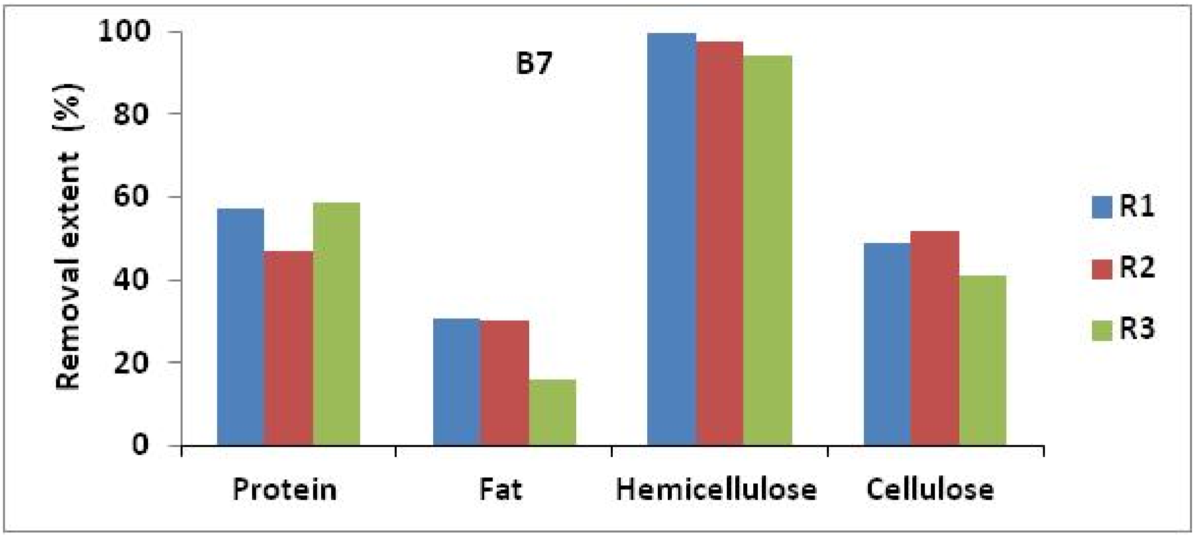

Figure S4 Degradation efficiency of the major components of restaurant food waste in R1, R2 and R3 during batch 7 (14-day SRT) of period 1

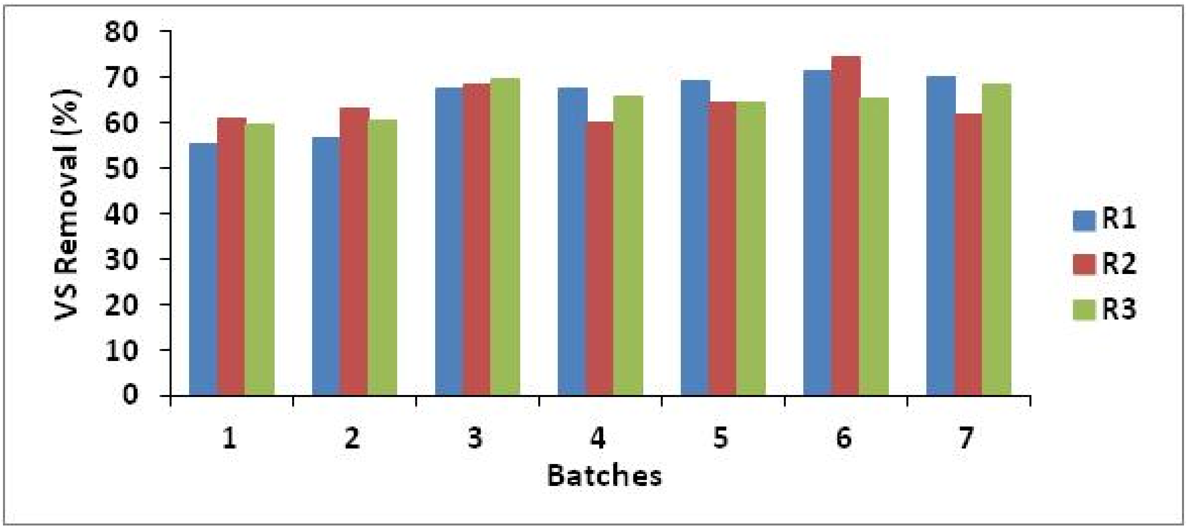

Figure S5 Volatile solid (VS) reductions from the restaurant food waste in R1, R2 and R3 operated at SRT of 14 days for each batch (period 1).

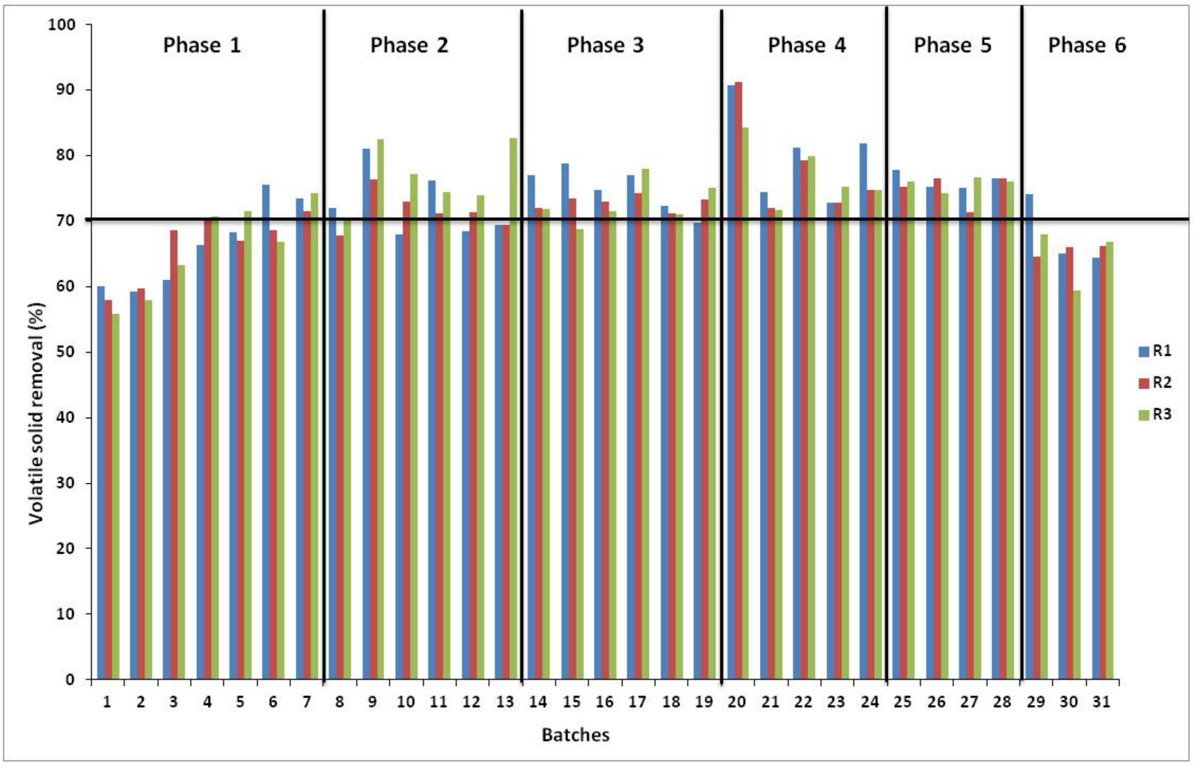

Figure S6 Volatile solid (VS) reductions from the restaurant food waste in triplicate reactors R1, R2 and R3 operated at 7-day SRT (period 2) for each batch. Phase 1: leachate was recirculated on top of solid bed once a day for 45 min; Phase 2: leachate recirculated four times per day; Phase 3: dilution of VFAs in starting liquid; Phase 4: bio-augmentation; Phase 5: dilution of VFA in the leachate on day 2; Phase 6: increase of loading rate.

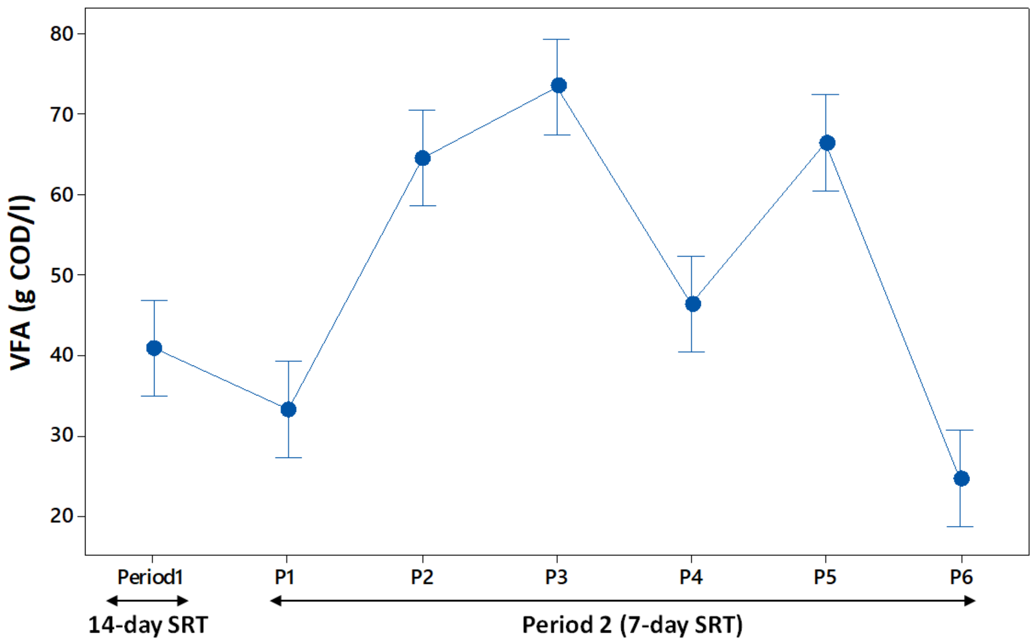

Figure S7 Plot of 95% confidence interval for mean of volatile fatty acid (VFA) concentration during period 1 and period 2.

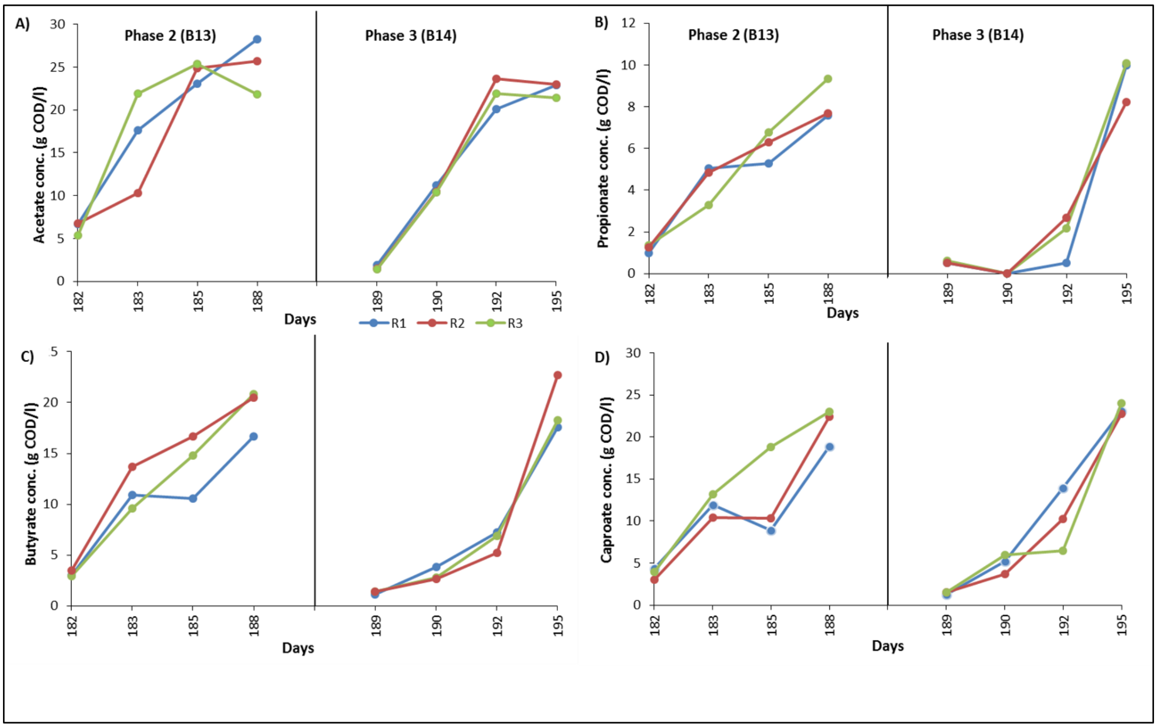

Figure S8 Profile of individual VFA production in leach-bed reactors R1, R2 and R3 during Phase 2 and 3 (period 2). Batch (B) 13 and 14 were selected to represent phase 2 and 3 corresponding to high and low VFA concentrations in starting liquid respectively. Data at each point represent average of duplicate measurements.

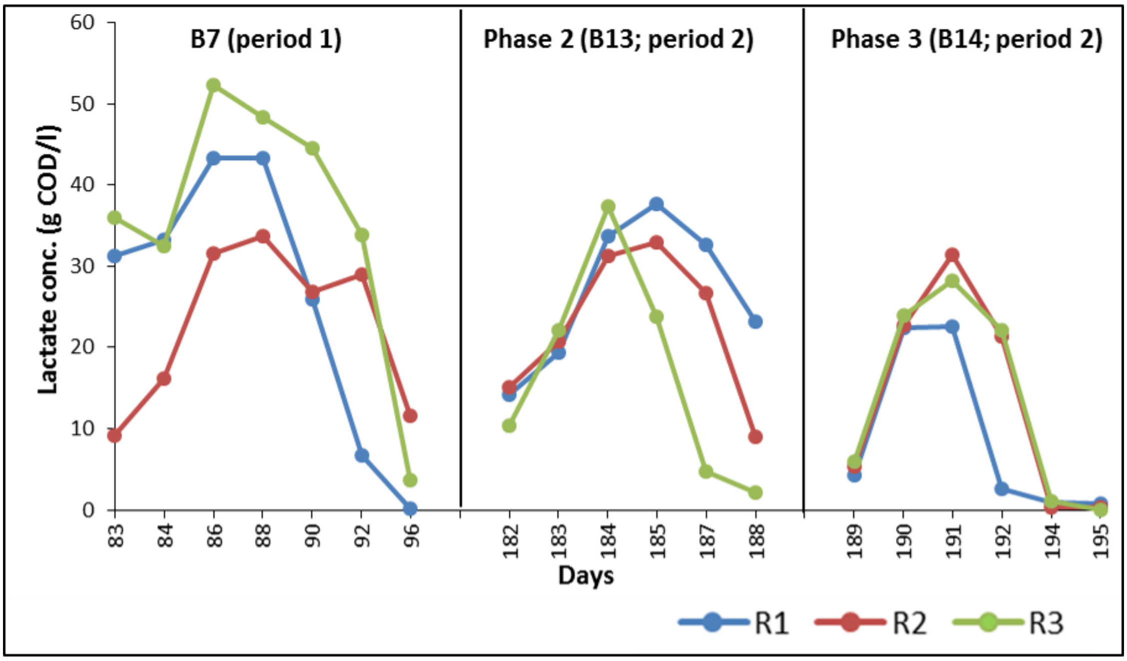

Figure S9 Profile of the lactate concentration in triplicate leach-bed rectors (R1 – R3) during period 1 and 2 (phase 2 and 3). Batch (B) 7 was selected to represent period 1 corresponding to 14-day SRT. B13 and 14 were selected to represent phase 2 and 3 corresponding to high and low VFA concentrations in starting liquid respectively. Value at each point is the average of duplicate measurements.

Table S2 Methane (CH_4) fraction of biogas during period 1(14-day SRT) and period 2 (7-day SRT) of reactor operation. Batch (B) 7 was selected to represent period 1while B5, 13, 14, 20, 27 and 31 represented period 2.

	**Period 1**	**Period 2**					
**Conditions**	**B7**	**Phase 1 B5**	**Phase 2 B13**	**Phase3 B14**	**Phase 4 B20**	**Phase 5 B27**	**Phase 6 B31**
CH_4 (% of biogas)	0.32 ± 0.5	5.81 ± 1.6	0.16 ± 0.3	1.5 ± 0.8	0.57 ± 0.3	1.75 ± 0.4	na

na: methane not detected in biogas

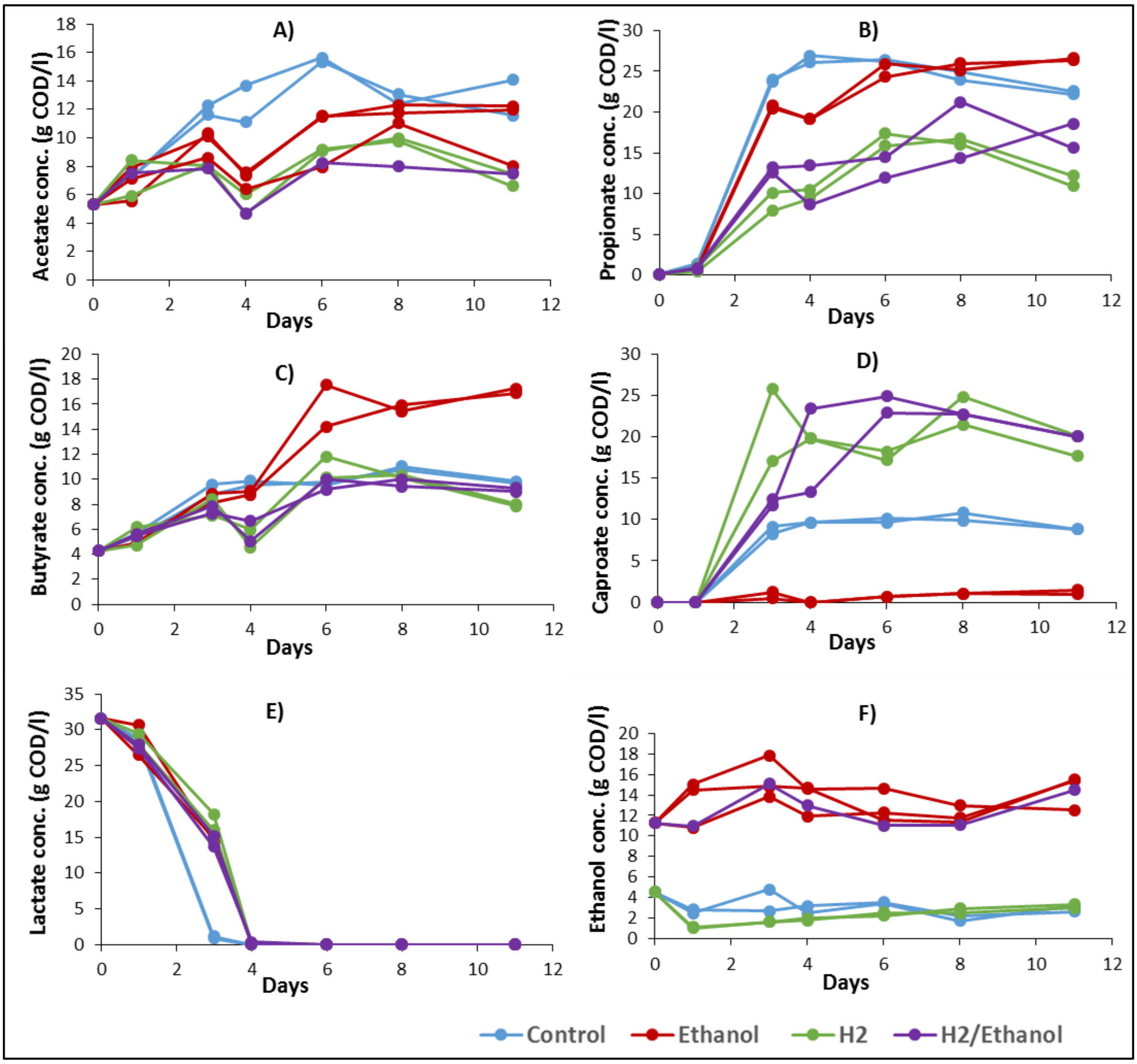

Figure S10 Production pattern for acetate (A) propionate (B), butyrate (C), caproic (D), lactate (E) and ethanol (F) obtained in the control, ethanol, hydrogen (H_2) and H_2/Ethanol supplemented vials during batch experiment using leach bed-reactor leachate. Data from the duplicate measurements are shown on the graph.

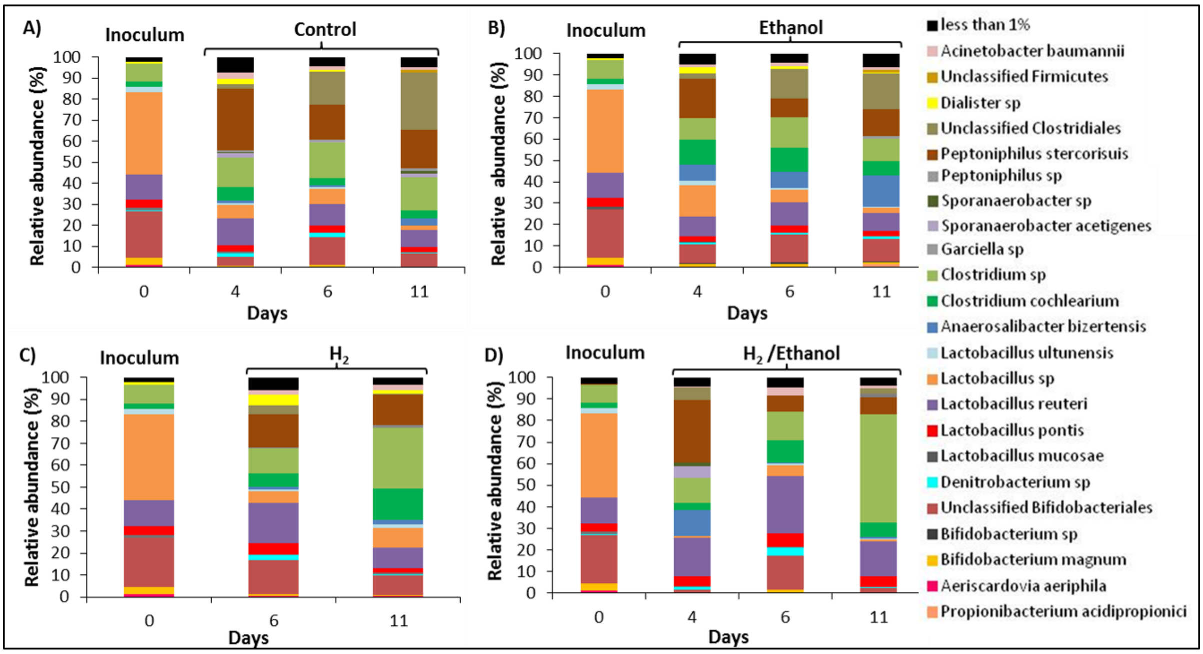

Figure S11 Taxonomic classification at species level of the 16S rRNA sequences showing microbial groups potentially involved in caproate production. Sequences were retrieved from Illumina MiSeq sequencing of cDNA samples generated from: A) control, B) ethanol, C) hydrogen (H_2) and D) hydrogen and ethanol (H_2/Ethanol) supplemented vials during batch experiments using leach-bed reactor leachate.

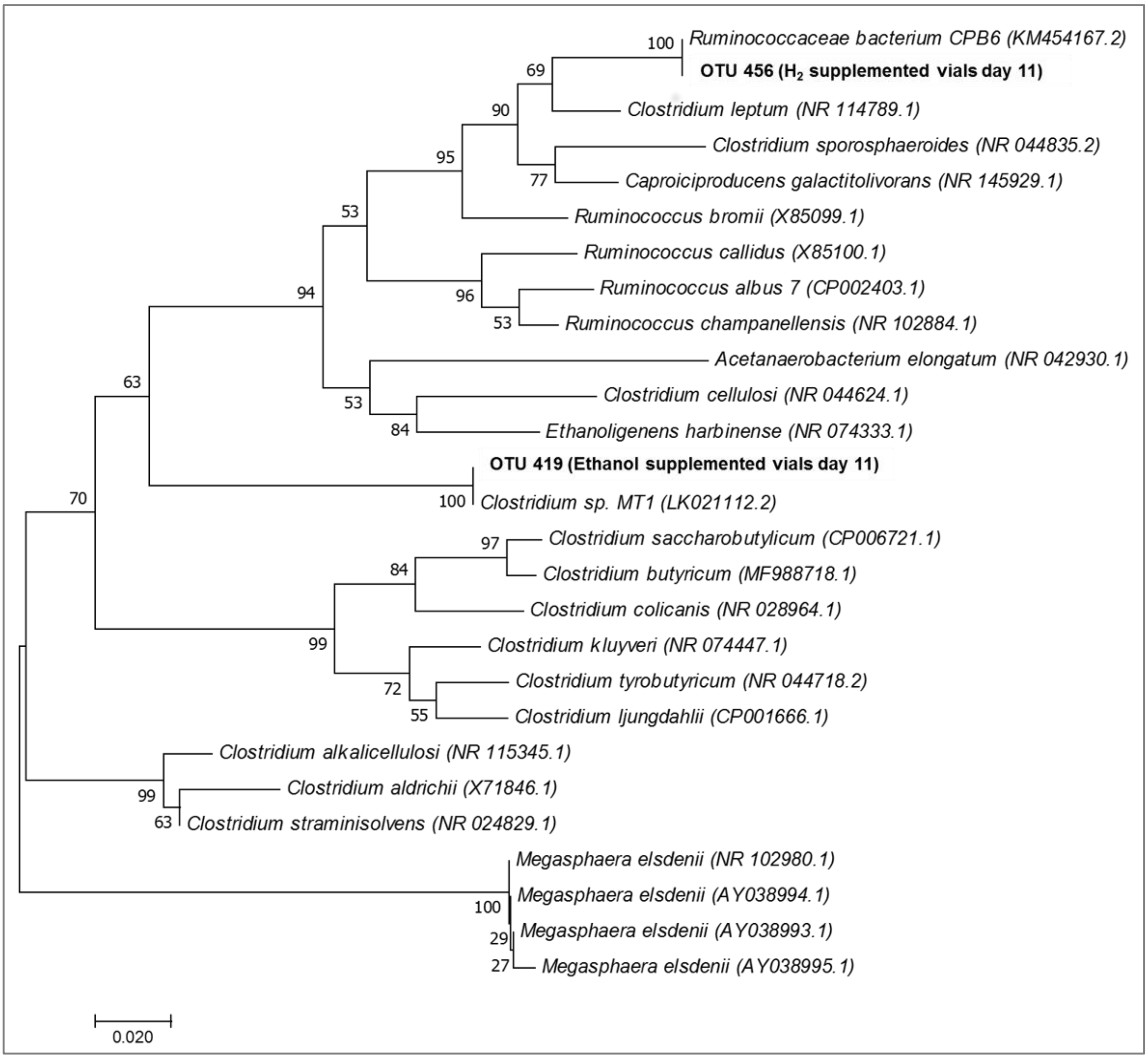

Figure S12 Phylogenetic tree build using the neighbor-joining method. Analysis performed using *16S rRNA* gene sequences. The numbers at the node represent the bootstrap values. The evolutionary distances were computed using the maximum composite likelihood method.

References

1. Angelidaki, I. and W. Sanders. Assessment of the anaerobic biodegradability of macropollutants. Re/Views in Environmental Science & Bio/Technology. 2004;3:117-129.
2. Folch, J., M. Lees, and G.H. Sloane Stanley. A simple method for the isolation and purification of total lipides from animal tissues. The Journal of biological chemistry. 1957;226:497-509.
3. Dubois, M., K.A. Gill, J.K. Hamilton, P.A. Rebers, and F. Smith. Colorimetric method for determination of sugar and related substances. Analytical Chemistry. 1958;28:350–356.
